# Decompensated Toxic Shock in a Gender-Diverse Adolescent: A Pediatric Emergency Medicine Simulation Case

**DOI:** 10.15766/mep_2374-8265.11615

**Published:** 2026-07-01

**Authors:** Taylor Freeburg, Todd Chang, Jack Brenner, Keya Manshadi

**Affiliations:** 1 Fellow, Division of Emergency Medicine and Transport, Children's Hospital Los Angeles; Simulation Affiliate, Las Madrinas Children's Hospital Los Angeles Simulation Center; 2 Attending Physician, Division of Emergency Medicine and Transport, Children's Hospital Los Angeles; Associate Professor of Pediatrics, Keck School of Medicine of the University of Southern California; Medical Director, Las Madrinas Children's Hospital Los Angeles Simulation Center; 3 Registered Nurse II, Division of Emergency Medicine and Transport, Children's Hospital Los Angeles; 4 Attending Physician, Division of Emergency Medicine and Transport, Children's Hospital Los Angeles; Clinical Assistant Professor of Pediatrics, Keck School of Medicine of the University of Southern California; Simulation Affiliate, Las Madrinas Children's Hospital Los Angeles Simulation Center

**Keywords:** Gender Diversity, Gender Equity, Trauma-Informed Care, Toxic Shock, Septic Shock, Pediatric Emergency Medicine, Simulation, Standardized Patient, Diversity, Equity, Inclusion, LGBTQ+ Health

## Abstract

**Introduction:**

Gender-diverse patients frequently encounter health care discrimination, contributing to delayed care and worse outcomes. Simulation offers a safe environment to integrate trauma-informed, gender-affirming care into high-acuity training. We developed a 1-hour simulation for pediatric emergency medicine (PEM) fellows combining management of toxic shock with affirming communication and trauma-informed practices.

**Methods:**

The case features a 13-year-old nonbinary patient presenting with abdominal pain, ultimately diagnosed with toxic shock from a retained tampon. A high-fidelity mannequin capable of intubation and genitourinary exams was used, with a standardized actor voicing the patient. Learners diagnosed and managed toxic shock including progression to decompensated shock requiring intraosseous access and endotracheal intubation. Performance was assessed using a 10-item critical actions checklist. Learners completed a postsimulation survey assessing confidence in providing gender-affirming care, use of trauma-informed communication, and perceived case effectiveness. The simulation was piloted with PEM fellows and is adaptable to pediatric or emergency medicine residents, attendings, and interprofessional teams.

**Results:**

Ten PEM fellows piloted the case. Across 2 sessions, learners completed 9/10 critical actions, with consistent omission of asking pronouns until prompted. All participants correctly diagnosed and managed toxic shock, with surveys indicating increased confidence in providing affirming, trauma-informed care. Seventy percent rated the case *very effective* and 30% rated it *extremely effective* in preparing participants to interact with patients using trauma-informed care principles.

**Discussion:**

This case demonstrates how high-stakes resuscitation training can incorporate equity-focused communication objectives. We offer a complete, educator-facing resource with case materials, script, debriefing guidance, and assessment tools to support adoption.

## Educational Objectives

By the end of this activity, learners will be able to:

1. Establish the patient's name and pronouns and incorporate them consistently throughout the simulation.
2. Perform a trauma-informed pelvic examination by explaining each step in advance, utilizing distraction strategies (such as allowing a support person or listening to music), and using the patient's preferred anatomical terminology.
3. Identify toxic shock by verbalizing more than 2 key clinical features (fever, rash, tachycardia, hypotension, and delayed capillary refill).
4. Manage toxic shock by ordering intravenous fluid resuscitation, antibiotics (including those that inhibit protein synthesis), and vasopressors for persistent hypotension.
5. Obtain intraosseous access when intravenous access is unsuccessful.
6. Intubate a patient demonstrating respiratory failure and a Glasgow Coma Scale score less than 8.

## Introduction

Gender-diverse patients often face significant barriers to care in emergency settings, including misgendering, inappropriate questioning, and both implicit and overt discrimination.^1,2^ These negative experiences can lead to delays in care, avoidance of the health care system, and increased risk of poor health outcomes.^3^ In contrast, when gender-diverse individuals perceive health care interactions as trauma informed, they report greater feelings of empowerment and emotional regulation, and reduced social withdrawal.^4^ The pediatric emergency department presents a critical opportunity to deliver affirming, trauma-informed care, particularly as it may be the first point of health care engagement for many youth.

Despite growing awareness of the importance of gender-affirming care, the literature demonstrates a consistent underexposure to sexual and gender minority health within graduate medical education settings.^5^ Emergency medicine physicians, in particular, receive limited formal instruction in this area.^6^ Through a systematic needs assessment guided by Kern's 6-step approach to curriculum development, our fellowship program identified a gap in its diversity, equity, and inclusion (DEI) curriculum and sought to address this gap through simulation-based education.

Simulation provides an ideal modality for teaching both procedural skills and interpersonal communication.^7^ Grounded in Kolb's Experiential Learning Theory, simulation allows for active, hands-on learning followed by reflective debriefing.^8^ Prior studies have explored simulation as a vehicle for DEI training, but few published simulations integrate trauma-informed communication within complex medical resuscitation.^9,10^ This blended approach is critical because it more accurately reflects the realities of clinical practice, where providers must balance the interpersonal and social complexities of each patient with the need for timely, high-stakes medical decision-making.

We developed and implemented a high-fidelity simulation case designed to teach pediatric emergency medicine (PEM) fellows both the recognition and management of toxic shock syndrome and the provision of trauma-informed, gender-affirming care. Our work builds on existing literature while offering a novel, blended approach to clinical and DEI-focused education. The case features a nonbinary adolescent presenting with abdominal pain due to a retained tampon who develops decompensated toxic shock as the case progresses. We piloted the case with our institution's PEM fellows and share both the educational design and implementation outcomes to guide adaptations at other institutions.

## Methods

### Development

We performed the pilot simulation in the Las Madrinas Simulation Center at Children's Hospital Los Angeles as part of a mandatory comprehensive simulation curriculum for PEM fellows. The learners were PEM fellows with prior pediatric residency training.

We designed the case utilizing Kern's 6-step approach to curriculum development. This simulation case was part of a larger effort to formally overhaul our fellowship's academic curriculum, which included restructuring a previously informal simulation program into a longitudinal, structured curriculum. The design team included PEM physicians, a pediatric resident, and an emergency department nurse. This interprofessional collaboration strengthened the educational design by incorporating diverse clinical and educational perspectives that improved the realism of the case.

To identify key resuscitation and procedural skills for this new simulation curriculum, we conducted both global and local needs assessments. The global needs assessment highlighted a need to improve care for gender-diverse patients. Locally, we used previously published key topics for PEM simulation-based curricula, developed through consensus techniques,^11^ as a framework for focus group discussions with program leadership and questionnaires for fellows and recent graduates. The focus group discussions identified several DEI-related educational priorities, including addressing bias, improving the use of respectful and appropriate language (particularly during challenging conversations), and optimizing care for patients with lower health literacy.

Although care of gender-diverse youths was not identified as a standalone domain in the local needs assessment, the themes of respectful communication, bias mitigation, and inclusive care were directly applicable. The decision to develop a case featuring a gender-diverse patient was informed by these broader DEI priorities and the authors’ specific interest in the care of gender-diverse youth. Gender diversity was further supported by the PEM fellowship leadership as a high-priority topic. Open-ended responses from stakeholders further supported inclusion of DEI content within the simulation curriculum. These needs assessments guided the selection of educational priorities best addressed through simulation.

Based on the needs assessment results, we incorporated toxic shock, intraosseous placement, and intubation into the case scenario alongside trauma-informed communication skills. A PEM physician and third-year pediatric resident developed the case and made iterative changes throughout its development. We consulted content experts from the Office of Opportunity, Integrity, and Impact and our pediatric emergency department to develop key learning objectives and to refine the case content, progression, and fidelity. Nonbinary colleagues provided feedback on the standardized actor script based on their lived experiences. We piloted the case with 3 third-year pediatric residents who provided real-time feedback, and we revised it accordingly. The case was then implemented with all 10 PEM fellows, divided into 2 groups of 5 who provided feedback in the form of postsimulation surveys. To ensure best practices in gender-affirming and trauma-informed care, we referenced UCSF Transgender Care Guidelines^12^ and the D-E-F Framework for Trauma-Informed Pediatric Care.^13^ We also referenced the Surviving Sepsis Campaign^14^ for best practices in managing septic shock. All case implementation materials, including the simulation script, structured debriefing guide, and other supporting materials, are included in the appendices. Generative AI (ChatGPT, OpenAI) was used to support Educational Summary Report manuscript editing for clarity and grammar. We take responsibility for the content modified by AI.

### Equipment/Environment

The full simulation case is presented in Appendix A, with a comprehensive list of equipment provided in Appendix B. We set up a simulated patient room in standard emergency room fashion and included monitors, oxygen delivery supplies, a pediatric code cart, intubation equipment, and an EZ I/O kit. We set the scene with Gaumard's Susie S901 mannequin^15^ with female pelvic anatomy dressed in a t-shirt and jeans with a chest binder and boxer underwear underneath lying on a gurney. The patient had 1 large-bore IV access connected to a drainage system that was hidden until requested by the participants. During the simulation introduction, participants were instructed to call the pharmacist for medications using the in-room telephone. All medications listed in Appendix B were not used but prepared in advance for variations in practice.

### Personnel

In each simulation, the learners were PEM fellows split into groups of 5. A PEM attending served as the facilitator, providing the introduction, delivering in-scenario guidance via microphone, and leading the debrief. A PEM nurse, who identifies as transgender, is a member of GLMA: Health Professionals Advancing LGBTQ+ Equality, and has led didactics on gender diversity in the workplace, provided real-time dialogue as the patient via an external speaker. A simulation technician managed vital signs, examination findings, and audiovisual support. A multidisciplinary team, including a PEM attending, a PEM nurse, and a pediatric resident with content expertise in toxic shock, trauma-informed care, and gender-affirming care, conducted the debriefing.

### Implementation

The simulation was conducted in a room at the simulation center, equipped with the aforementioned equipment (see Appendix B). The facilitator accompanied the learners to the simulation room and informed them that they were going to evaluate a 13-year-old previously healthy adolescent who presents to the emergency department with 2 days of fever and abdominal pain. The facilitator then observed the simulation through a 1-way mirror and via audiovisual recording from a control room with the simulation technician, a standardized actor, and another co-facilitator. During the simulation, the facilitator used a microphone to communicate additional relevant information and played the role of the pharmacist via telephone. The standardized actor voiced the patient via a pillow speaker following a script (Appendix C). A second facilitator displayed laboratory and imaging results (Appendix D) via in-room monitors. After obtaining the initial history, exam, and work-up, the facilitator fast-forwarded the simulation 60 minutes to when the patient clinically deteriorates. The simulation took approximately 20 minutes to complete. Immediately following the simulation, learners participated in a 30-minute debrief (Appendix E).

### Debriefing

We used the PEARLS Healthcare Debriefing Tool to guide debriefing.^16^ The facilitator began by eliciting emotional reactions to explore initial feelings. The facilitator then asked the learners to summarize the simulation to create a shared mental model. This was followed by a review of the learning objectives and an open discussion focused on both clinical management and trauma-informed, gender-affirming communication. Example discussion questions included “How did the patient's identity differ from what was listed in the electronic medical record?” and “How did you feel seeing the patient distressed by the idea of having to have a physical exam, yet knowing you had to do it for their safety?” Additional discussion topics focused on clinical management included toxic shock, pulmonary edema and respiratory failure, and intraosseous placement. Complete debriefing materials are provided in Appendix E.

### Assessment

The facilitator assessed learner behaviors (Kirkpatrick level 3) using a critical actions checklist (Appendix F), which was designed to capture observable, objective actions that learners were expected to complete during the scenario. The checklist items were derived from the case learning objectives and reviewed by faculty experts in PEM, toxic shock management, and gender-affirming care. Learners also received formative feedback on the case during the 30-minute debriefing. In addition, learners provided feedback on the pilot testing through a postsimulation survey that assessed learners’ self-reported knowledge, confidence, and perception of the learning objectives (Kirkpatrick level 1). Following the debriefing, learners were provided with a QR code to complete this optional anonymous Microsoft Forms feedback survey without research staff present. Taylor Freeburg and Keya Manshadi independently reviewed open-ended survey responses and identified recurring themes through an iterative discussion process. We provide a Microsoft Word document version of this survey in Appendix G.

The Children's Hospital Los Angeles Institutional Review Board reviewed this project and designated it as exempt (CHLA-24-00343). Participation in the postsimulation survey was voluntary and anonymous, and completion of the survey implied consent.

## Results

Ten PEM fellows participated in this simulation in January 2025. The same case was run twice, each with a group of 5 fellows.

When piloted with 10 fellows, learners reliably completed 9 of 10 critical action items. All participants correctly diagnosed toxic shock and appropriately managed decompensated shock, including intraosseous access and intubation, when needed. The consistently missed action item across both groups was asking for the patient's pronouns.

All 10 fellows completed the implementation feedback survey. Eighty percent (8 out of 10) of participants *strongly agreed*, and 20% (2 out of 10) *agreed* that the simulation increased their knowledge of caring for transgender patients. All participants rated the simulation as effective in preparing participants to interact with patients using trauma-informed care principles, with 70% (7 out of 10) rating it *very effective* and 30% (3 out of 10) rating it *extremely effective*. One hundred percent (10 out of 10) of participants reported that the simulation case was effective in teaching recognition and management of toxic shock. Complete quantitative results of the feedback survey are included in the Table.

**Table.**
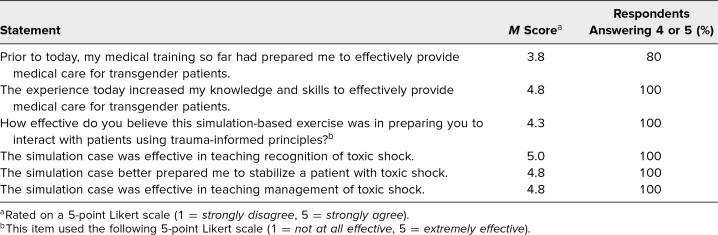
Participant Feedback on the Simulation in Relation to Learning Objectives (*N* = 10)

Analysis of open-ended survey responses revealed key takeaways. When asked what participants found most useful from the session, 4 learners highlighted the importance of language and pronoun use, whereas 3 learners emphasized insights gained around trauma-informed care. One participant noted the simulation helped identify “ways to make patients feel more comfortable,” and another brought to attention the concept of “being mindful of gender pronouns in a critical situation.”

When asked how they would apply their learning in future clinical encounters, 5 learners indicated plans to include their pronouns when introducing themselves to patients. One participant shared an intention to “acknowledge out loud when I make a gender label mistake,” whereas another described “coming up with a standard way to introduce myself/pronouns as a method to consistently segue into asking for patients’ preferred names/pronouns.”

## Discussion

This educational intervention targeted for PEM fellows involved diagnosing and managing decompensated toxic shock in a nonbinary adolescent. Feedback from the implementation survey revealed that learners found the simulation effective in teaching recognition and management of toxic shock, as well as trauma-informed principles. In addition, all participants *agreed* that the simulation increased their knowledge in caring for transgender patients.

This simulation effectively combines high-stakes resuscitation training with equity-focused communication and adds to a gap in the current educational literature by focusing on a gender-diverse adolescent. During the development of this educational intervention, transgender and nonbinary identified educator perspectives were prioritized to ensure that lived experience was prioritized, valued, and honored in the finished product. In addition, the case integrates trauma-informed care even as the patient decompensates, so learners can practice trauma-informed principles in high-stress situations.

Based on the results of the critical actions checklist, all learners correctly diagnosed and managed decompensated toxic shock; however, they did not consistently use correct pronouns. Learners initially failed to ask the patient's pronouns, but after prompting from the standardized actor, they adjusted their language appropriately. As the patient's condition deteriorated, learners occasionally reverted to the incorrect pronouns listed in the electronic medical record. In the postsimulation survey, learners reported increased knowledge and confidence in caring for gender-diverse patients and commented that in future clinical encounters, they would include their pronouns when introducing themselves to patients. This suggests that the educational intervention was effective in increasing awareness of pronoun use and in providing a safe space to explore and improve communication practices.

During the debrief, learners acknowledged that while they often remember to ask about pronouns in stable situations, the stress of managing a critically ill patient challenged their ability to maintain this practice. This observation mirrors findings from the broader literature showing that high cognitive load in PEM contributes to medical errors and lapses in best practices.^17,18^ In keeping with Kolb's Experiential Learning Theory, learners identified strategies such as incorporating pronouns into their standard introductory script when entering a patient's room or updating the electronic medical record with the patient's pronouns so that gender-affirming language becomes routine and therefore easier to sustain, even in high-stakes clinical scenarios. Our findings underscore the ongoing need for training in gender-affirming communication, particularly in critical scenarios where competing priorities may make affirming practices more difficult to sustain.

With regard to the trauma-informed care objectives, although both groups ultimately performed the genitourinary exam using trauma-informed techniques, including offering the presence of a support person or Child Life staff and using active distraction, both groups required prompting from the standardized actor to initiate the exam. This behavior reveals hesitation in applying these skills independently. In the debriefing, learners acknowledged the importance of the exam but were hesitant to initiate it due to assumptions that another team member would perform it, competing clinical priorities, or uncertainty about how to approach it in a gender-diverse patient. Similarly, prior studies have shown that emergency medicine physicians often delay or avoid performing genitourinary exams due to similar discomfort and assumptions.^19,20^ These parallels suggest that while learners grasp trauma-informed principles, additional training is needed to strengthen confidence and decision-making around initiating sensitive exams.

This simulation was created for PEM fellows but can be easily applied to multidisciplinary teams (with the addition of attending physicians, nurses, respiratory therapists, pharmacists, and/or Child Life staff). In addition, the learning objectives can be adapted for pediatric, family medicine, or emergency medicine residents. In lower-resource learning settings or when adapting the simulation for pediatric or family medicine residents, educators could use the first of the case materials to model a gender-affirming, trauma-informed pelvic exam without the subsequent clinical decline, removing the need for intraosseous access and intubation. Additionally, we acknowledge that laws and limitations regarding gender-affirming care vary from state to state. This case was purposely designed to include the provision of emergency care with equity-focused communication skills and does not specifically address gender-affirming treatments to allow for use in states regardless of laws surrounding gender care.

This simulation had several limitations. First, the high-fidelity mannequin could not replicate the motor findings needed to accurately determine a Glasgow Coma Scale and therefore could not fully represent altered mental status. Additionally, learners noted in the debriefing that interacting with the standardized actor through the mannequin limited their ability to form an emotional connection with the patient, which may have contributed to delays in asking about pronouns. In future iterations of this simulation, we plan to have the standardized actor in the same room as learners to both encourage the emotional connection with learners and simulate the patient's declining mental status with task trainers for the pelvic exam, intraosseous access, and intubation portions of the case.

Second, the study's results are limited by the small sample size. As this simulation was a pilot study, we only included participants from a single PEM fellowship program. Expanding the simulation across multiple institutions would increase sample size and generalizability. Repeating the case annually at a single institution could also reinforce learning and allow for assessment of long-term knowledge retention. The trauma-informed care and gender-affirming communication learning objectives and debriefing outline could be applied to other existing high-acuity emergency medicine simulations to further reinforce these skills in other clinical situations.

In conclusion, we designed and piloted a simulation that addresses both resuscitation and DEI-focused communications learning objectives. Although the study was piloted with PEM fellows, the simulation can be adapted for a larger audience, including pediatric or emergency medicine residents, faculty, or multidisciplinary teams. Our implementation outcomes support the case's feasibility and educational value. This simulation case's blended approach mirrors the complexity of real-world emergency medicine, where physicians must balance acute resuscitation with sensitive, patient-centered communication. Simulation provides a safe environment to practice these skills until they become routine, supporting the delivery of affirming, trauma-informed care in real-world emergency settings.

## Appendices


Simulation Case.docxSimulation Case Equipment.docxStandardized Actor Script.docxCase Materials.pptxDebriefing Outline.docxCritical Actions Checklist.docxPostsimulation Survey.docx
*All appendices are peer reviewed as integral parts of the Original Publication.*
