# Supplementary material for: Decompensated Toxic Shock in a Gender-Diverse Adolescent: A Pediatric Emergency Medicine Simulation Case
Source: MedEdPORTAL. 2026 Jul 1;22:11615. doi: 10.15766/mep_2374-8265.11615 (PMC13319108; doi:10.15766/mep_2374-8265.11615)
Supplement: Supplementary file 1 — Simulation Case.docxSimulation Case Equipment.docxStandardized Actor Script.docxCase Materials.pptxDebriefing Outline.docxCritical Actions Checklist.docxPostsimulation Survey.docx [file mep_2374-8265.11615-s001.zip › A. Simulation Case.docx]

Appendix A: Simulation Case

*Instructions: This document contains the complete simulation case, including the case stem, scenario progression, expected learner actions, and facilitator prompts. Facilitators and operators should review this document in advance to familiarize themselves with the case flow, key decision points, and clinical deterioration timeline. During the simulation, facilitators should guide progression using the provided cues and adjust pacing based on learner performance while ensuring that core learning objectives are addressed.*

| SIMULATION CASE TITLE: Decompensated Toxic Shock in a Gender Diverse Adolescent: A Pediatric Emergency Medicine Simulation Case  AUTHORS: Taylor Freeburg, Keya Manshadi  LEARNER AUDIENCE: Pediatric Emergency Medicine Fellows | |
| --- | --- |
| PATIENT NAME: Jessie (legal name in medical record: Jessica)  PATIENT AGE: 13 years old  CHIEF COMPLAINT: fevers, abdominal pain, rash  PHYSICAL SETTING: pediatric emergency room | |
|  | |
| Brief Narrative Description of Case | Jessie (legal name in the medical record is Jessica), a 13-year-old, non-binary (they/them) adolescent assigned female at birth, presents to the emergency department with fevers and abdominal pain found to be in toxic shock secondary to prolonged tampon use requiring vasoactive support and PICU admission. |
| Primary Learning Objectives | 1. Establish the patient’s name and pronouns and incorporate them consistently throughout the simulation. 2. Perform a trauma-informed pelvic examination by explaining each step in advance, utilizing distraction strategies (such as allowing a support person or listening to music), and using the patient’s preferred anatomical terminology. 3. Identify toxic shock by verbalizing > 2 key clinical features (fever, rash, tachycardia, hypotension, and delayed capillary refill). 4. Manage toxic shock by ordering intravenous fluid resuscitation, antibiotics (including those that inhibit protein synthesis) and vasopressors for persistent hypotension. 5. Obtain intraosseous access when intravenous access is unsuccessful. 6. Intubate a patient demonstrating respiratory failure and a Glasgow Coma Scale score <8. |
| Critical Actions | - Perform GU exam/remove tampon   - If they fail to do a GU exam after the first bolus of fluids is given, the patient states: “My tampon has been stuck for four days, and I can’t get it out” - Obtain I/O access after initial IV access lost   - Nurse states: “It doesn’t look like the IV team can get access. What alternatives do you recommend?”   - If a central line is requested, the nurse states: “We don’t have the supplies, but we have an IO kit.” - Recognize shock   - If they fail to start fluid resuscitation, HR elevates to 180 and BP drops to 75/35 - Recognize pulmonary edema and respiratory failure   - If they fail to intubate, the nurse states: “Their oxygen saturation remains low with oxygen support. What alternatives do you recommend?” |
| Learner Preparation or Prework | No pre-work or preparation |

| Initial Presentation | | | |
| --- | --- | --- | --- |
| Initial Vital Signs | T 38, HR 110, BP 100/60, RR 26, SpO2 99 | | |
| Overall Setting and Appearance | The room is set up as a standard Emergency Department patient room (per institution). Available within the room is a standard vital sign monitor, oxygen delivery supplies, and a phone or other method to call consultants. Within, or just outside, the room there is a code cart.  The following items should also be available in the room if not already: 1) Three-way stopcock (for “push-pull” administration of fluids) 2) EZ-IO kit (needles, power driver, stabilizer dressing and extension set) 3) Intubation equipment (video laryngoscope and/or direct laryngoscope, endotracheal tubes, stylets and CO2 detector).  The mannequin (adolescent with female pelvic anatomy) is on a gurney in the room, has short hair and is dressed in a t-shirt, jeans, and underwear. There is diffuse erythroderma of the mannequin’s skin. There is a tampon covered with green/red discharge placed within the vaginal opening of the mannequin. A pillow speaker (or other speaker device) should be available to allow for mannequin speech. | | |
| Standardized Participants (and Their Roles in the Room at Case Start) | *Facilitator reads case stem:* A 13-year-old previously healthy adolescent presents to the emergency department with two days of fever, muscle aches, abdominal pain, watery diarrhea, and vomiting. The residents are busy seeing other patients, so the ED attending asks you to obtain the initial history and examine the patient.  Standardized actor (SA), voicing the mannequin via pillow speaker (See Appendix D for full script and prompts). SA should be played by someone with knowledge and experience with gender diversity, gender care, and trauma informed care.  SA is a 13-year-old presenting to the emergency department for 2 days of abdominal pain and fevers. They got their first period 4 days ago and inserted a tampon to stop the bleeding. However, the tampon became stuck and has remained in place for the past 4 days. They came to the ED on their own because they were embarrassed to tell their family.   1. When the medical team enters the room: “Who are you?” 2. HPI questions (see below) 3. If the learner asks name and/or pronouns:    1. “Jessie” / “They/them” 4. If the learner does not address SA:    1. “What’s going on?”    2. “Can you make my belly pain stop?” 5. If the learner misgenders SA or calls SA Jessica (including during communication with other team members), then SA becomes angry and says:    1. “I don’t go by Jessica.” When asked what they go by: “Jessie”    2. “You can’t call me she.”    3. “Stop calling me that.”    4. “I don’t use that.” 6. If learner uses appropriate language:    1. SA is calm, relaxed, and cooperative. Provides less abrupt responses when asked. 7. During genitourinary exam:    1. SA should adjust your level of anxiety based on how sensitive or gentle the trainee is.    2. If the trainee is abrupt, not talking through all the steps, or not recognizing SA’s baseline anxiety, SA should have increased anxiety and impede the trainee’s ability to perform the exam.    3. If the trainee is calm, professional, gentle, and sensitive, then SA should act visibly more relaxed. 8. When the facilitator reports that “60 minutes have passed” (signaling progression of the case):    1. SA should become more disoriented throughout the remainder of the case. May act more confused and require prompting multiple times when asked questions. | | |
| HPI | All questions must be asked by the learners to receive appropriate responses. See Appendix D for detailed information.  Purpose of the visit: “My belly hurts, and I have a fever.”  If the learner asks for more details: “The fevers and belly pain started two days ago. It’s the worst pain I’ve ever had, and I haven’t felt like eating anything for the last day.”  If asked about fevers: “My temperature was 102.1F yesterday, my whole body hurts, and I have chills.”  If asked about rashes: “The rash started yesterday. It doesn’t itch. It’s not painful.”  If asked about abdominal pain: “My lower belly hurts, and the pain is getting worse. I started throwing up yesterday and don’t feel like eating anything.”  If asked about pain while urinating: “I don’t have pain when I pee, but there is a smelly green discharge in my underwear.”  If asked about your periods: “I started bleeding for the first time 4 days ago. I tried using a tampon, but it got stuck, and I don’t know how to get it out.”  All other systems or diseases are negative.  If asked where you live: “I live at home with my parents. I took the bus to the ED alone because I was embarrassed and didn’t want my parents to worry.”  If asked about drugs/alcohol: “I have never tried any drugs or alcohol”  If asked about sexual history: “I have never been sexually active.” | | |
| Past Medical/Surgical History | Medications | Allergies | Family History |
| Previously healthy. No prior surgeries. | Does not take any medications at home. | NKDA | No sick contacts. |
| Physical Examination | | | |
| General | Awake, uncomfortable, diaphoretic | | |
| HEENT | Dry lips. Pupils are equal and reactive. Red lips/tongue. | | |
| Lungs | Mildly tachypneic. Clear lungs. | | |
| Cardiovascular | Mild tachycardia. | | |
| Abdomen | Soft, non-distended. Tender to palpation in lower quadrants and suprapubic regions. | | |
| Neurological | GCS 15 | | |
| Skin | Flushed. Diffuse erythematous macular rash. | | |
| GU | Normal female pelvic anatomy with tampon covered in green mucopurulent discharge in vaginal orifice. | | |

| Instructor Notes - Changes and CASE Branch Points | | | |
| --- | --- | --- | --- |
| Time Point / State | Expected Participant Actions, Patient Response to Intervention and Triggers to Progress | Patient Status and Operator Prompts | Monitor (Vital Signs) |
| 0:00 minutes  Initial Presentation | Trigger: Learners enter the room to assess the patient. | Gen: Awake, uncomfortable, and diaphoretic.  HEENT: Dry lips, pupils equal and reactive, red lips and tongue.  Neuro: Alert, GCS 15.  Resp: Tachypnea, clear lungs.  CV: Tachycardia, regular rhythm, no murmurs.  GI: Soft, non-distended, +tender in lower quadrants and suprapubic region.  GU: Female pelvic anatomy with tampon covered in green/red mucopurulent discharge in vaginal orifice.  Skin: Flushed, diffuse erythematous macular rash. | T: 38C HR: 110 BP: 100/60  RR: 26 SpO2: 99% |
| 00:30 minutes  Initial History | Trigger: Learner begins to ask about the initial history. | No Change in Status  *See Actor Script* | Unchanged |
| 02:00 minutes  Initial Exam | Trigger: Learner begins to conduct physical exam  Expected Interventions:  1) Learner to obtain assent to perform GU exam 2) GU exam performed. See the actor's script for details. Briefly: If the learner does not use trauma informed care principles / techniques, the patient is fearful and does not cooperate. | No Change in Status  *See Actor Script* | Unchanged |
| 04:00 minutes  Initial Work-up and Management | Trigger: Learner begins work-up / orders OR Bedside RN prompts: “What work-up are you planning to order.”  Expected Interventions:  1) If antipyretics given: T 38C → 37C HR 110 → 95 (over 30s)  2) If IV requested:  Successful → IV uncovered  3) If labs/imaging ordered:  Bedside RN: “Great. I’ll get those started.” | No Change in Status | Unchanged |
| 05:00 minutes  Fluid Refractory Septic/Toxic Shock | Trigger: Facilitator states: “You continue to see other patients while waiting for labs. Sixty (60) minutes have now gone by.”  Bedside RN: “Can you come back to evaluate this patient? They are tachycardic and do not look well.”  Labs/imaging return:  1) CBG: Slide 3  2) Urine: Slide 4  3) CBC: Slide 5  4) CMP, CRP: Slide 6  5) PT/PTT/INR: Slide 7  6) Chest XR and Abdominal XR: Slide 8 (tampon not removed) or Slide 9 (tampon removed)  7) Abdominal US: Slide 10  Expected Interventions:  1) *Isotonic fluid bolus #1 delivered:  HR no change  RR 30 → 34  SpO2 99% → 97%  2) *Isotonic fluid bolus #2 delivered:  HR no change  RR 34 → 40  SpO2 97% → 92%  Crackles develop  3) May order PRBCs, platelets, FFP:  No change in status  4) Antibiotics ordered:  No change in status  *When a fluid bolus is ordered or if administered via push-pull technique, the facilitator states “20 minutes have now gone by. You have finished giving the full bolus and the vital signs reflect your intervention.” | Gen: More disoriented and less direct in interaction. Requires prompting twice for some questions, but still responsive.  HEENT: Unchanged  Neuro: Disoriented/confused and more fatigued. Eyes closed (opens to sound). GCS 12 (E3, V4, M5)  Resp: Tachypnea. Crackles develop (if 2+ boluses given)  CV: Tachycardia. Bounding pulses. Flash capillary refill.  GI: Unchanged  GU: Unchanged  Skin: Unchanged | T: 37C  HR: 160  BP: 80/50  RR: 34  SpO2: 99% |
| 07:00 minutes  Fluid Refractory Septic/Toxic Shock with Pulmonary Edema and Initiation of Pressor Support | Trigger: Second isotonic fluid bolus given  Expected Interventions:  1) Isotonic fluid bolus #3 given:  HR 155 → 165  SpO2 92% → 84%  2) Oxygen applied:  SpO2 92 → 93%  3) Repeat chest XR: Slide 11  4) Epinephrine or norepinephrine started:  HR 155 → 140  BP 80/50 → 95/60  *When a fluid bolus is ordered or if administered via push-pull technique, the facilitator states “20 minutes have now gone by. You have finished giving the full bolus and the vital signs reflect your intervention.” | Gen: Unchanged  HEENT: Unchanged  Neuro: Unchanged  Resp: Tachypnea. Crackles in all fields. +retractions  CV: Unchanged  GI: Unchanged  GU: Unchanged  Skin: Unchanged | T: 37C  HR: 155  BP: 80/50  RR: 40 SpO2: 92% |
| 08:00 minutes  Loss of IV Access, Worsening Mental Status and Respiratory Failure | Trigger: 1 minute after pressors started  Facilitator: “IV access is lost.”  Expected Interventions:  1) IO attempted:  Successful  2) Intubation:  Successful. SpO2 93 -> 98%  3) Re-initiation of pressors  HR 155 → 140  BP 80/50 → 95/60 | Gen: Becomes less responsive. Intermittently groans to pain/stimulation. Does not answer questions.  HEENT: Unchanged  Neuro: GCS 8 (E2, V2, M4)  Resp: Bradypnea. Crackles in all fields. +retractions  CV: Unchanged  GI: Unchanged  GU: Unchanged  Skin: Unchanged | T: 37C  HR: 155 BP: 80/50 RR: 14  SpO2: 93% |
| 12:00 minutes  Stabilization and Transfer of Care | Trigger: IO access established, patient intubated and pressors restarted.  PICU team arrives at beside and requests sign-out  Expected Interventions:  1) Handoff given:  Case ends | No Change in Status | T: 37C  HR: 140 BP: 95/60 RR: ventilated  SpO2: 98% |

Ideal Scenario Flow

Learners enter the room and immediately introduce themselves to the patient and ask the patient for their name and pronouns. The standardized actor responds with “Jessie…they/them,” and the team consistently incorporates this information throughout the encounter. The medical team quickly places Jessie on the bedside monitor and performs an initial assessment. They obtain a focused history and exam using trauma informed communication, validating Jessie’s concerns, and explicitly explaining each step before proceeding.

The team recognizes that Jessie is febrile and tachycardic, and they have a diffuse erythematous rash with abdominal pain and a retained tampon. Integrating this information, the learners make the diagnosis of toxic shock syndrome and promptly initiate a sepsis bundle. They immediately establish IV access, draw labs, provide broad-spectrum antibiotics including clindamycin for toxin suppression, administer an isotonic fluid bolus, and remove the retained tampon. Laboratory studies obtained include a CBC with leukocytosis and thrombocytopenia, a blood gas with elevated lactate, a chemistry with mildly elevated liver enzymes, BUN/Creatinine and CRP, and a coagulation panel with elevated D-dimer, a negative urine pregnancy, a urine analysis with moderate blood but without leukocyte esterase or nitrites. The ultrasound of the abdomen demonstrated a normal appendix and ovaries.

Learners reassess Jessie frequently, noticing worsening tachycardia and mental status after fluid administration. They correctly identify fluid-refractory shock and the early evolution of pulmonary edema, pausing further boluses and initiating vasoactive support. When IV access is lost, the team immediately recognizes the need for rapid alternative access and places an intraosseous line.

As Jessie becomes more confused and hypoxic, the learners recognize impending respiratory failure and prepare for intubation. A chest x-ray is obtained and demonstrates increased infiltrates and pulmonary edema. Learners pre-oxygenate, select appropriate weight-based rapid sequence intubation medications and equipment, and successfully intubate Jessie.

Once stabilized with an endotracheal tube, IO access, antibiotics and a vasoactive infusion, learners reassess the response to interventions and determine the need for any other evaluation or management. The PICU team arrives, and the learners provide a concise and complete handoff to the ICU team, including interventions performed, response to therapy and outstanding concerns.

Anticipated Management Mistakes

1. Misgendering or inconsistent use of pronouns
   1. Learners may fail to ask Jessie’s pronouns or may inconsistently use them during the encounter. The standardized actor has scripting prompts so that if misgendering occurs, the actor will bring this to the learner’s attention. The standardized actor will mirror the learner’s empathy and ability to use correct pronouns, with more frustration / lack of cooperation when being misgendered and calm / cooperativity when appropriate language is used.
   2. Some learners noted that interacting with the standardized actor through the mannequin limited their ability to form an emotional connection with the patient, which may have contributed to delays in asking about pronouns. Consider adapting the simulation to have the standardized actor present in the same room as learners, reserving the mannequin for invasive procedures (pelvic exam, intraosseous access, and intubation).
2. Not performing a genitourinary exam early in the evaluation
   1. Learners may delay or avoid the GU exam despite red flags in the history. This often results in missed identification of the retained tampon and delays definitive management. The standardized actor script has scripted escalation to reveal the tampon if learners fail to perform a GU exam. In addition, two abdominal x-rays are provided in the supplemental material section, allowing facilitators to share an x-ray with and without a tampon inserted, to provide a subtle clue that a GU exam may be needed.
3. Not using trauma-informed techniques during the GU exam
   1. Learners may proceed abruptly, fail to explain steps, or overlook Jessie’s anxiety when it comes to performing the GU exam. The standardized actor is scripted to give learners pause if proceeding without using trauma-informed techniques. This portion of the simulation allows the facilitator / debriefer to guide discussions on consent, pacing, and trauma-informed language in the emergency room setting.
4. Delayed recognition of toxic shock syndrome
   1. Learners may anchor on gastroenteritis, appendicitis, urinary tract infection, or other infectious causes of the patient’s symptoms, missing the diagnosis of toxic shock syndrome. As a result, there may be delays in fluid resuscitation, antibiotic administration (or correct antibiotic choice), and/or escalation of pressors.
5. Over-aggressive fluid resuscitation despite signs of pulmonary edema
   1. Learners may continue to give fluid boluses without reassessing lung exam or oxygenation, leaning to worsening respiratory compromise. This case intentionally presents crackles and hypoxia after the second bolus to cue learners to shift towards vasoactive support. Further vital sign changes, such as worsening hypoxia and tachycardia, are also meant to help guide learners.
6. Ineffective management after loss of IV access
   1. Learners may not recognize IO access as the preferred mode of access in a critical patient without IV access. The facilitator may choose to prompt the learner if a delay is noted, or incorrect access method is requested by the learners.
7. Delayed recognition of impending respiratory failure
   1. Learners may not recognize the worsening hypoxemia, tachypnea and subsequent bradypnea or increased crackles as a sign of impending respiratory failure. The case is designed to allow for continued deterioration until the team proactively recognizes this and manages the airway.
