# Supplementary material for: Decompensated Toxic Shock in a Gender-Diverse Adolescent: A Pediatric Emergency Medicine Simulation Case
Source: MedEdPORTAL. 2026 Jul 1;22:11615. doi: 10.15766/mep_2374-8265.11615 (PMC13319108; doi:10.15766/mep_2374-8265.11615)
Supplement: Supplementary file 1 — Simulation Case.docxSimulation Case Equipment.docxStandardized Actor Script.docxCase Materials.pptxDebriefing Outline.docxCritical Actions Checklist.docxPostsimulation Survey.docx [file mep_2374-8265.11615-s001.zip › B. Simulation Case Equipment.docx]

**Appendix B: Simulation Case Equipment**

*Instructions: This document provides a comprehensive list of equipment required to run the simulation. Facilitators and simulation staff should use this list to prepare the environment in advance. Equipment may be adapted based on local availability while maintaining the core components necessary to achieve the learning objectives.*

*In lower-resource settings, modifications may include having the standardized actor present in the room with learners rather than using a microphone or speaker system and substituting a task trainer for invasive procedures in place of a high-fidelity mannequin. If intravenous access to a drainage system is not available, learners may verbalize medication and fluid administration. These adaptations preserve key learning objectives while allowing flexibility in implementation.*

| **Mannequin** | Gaumard Susie^Ⓡ^ S901 or similar mannequin representative of an adolescent. The mannequin should have female pelvic anatomy, be capable of intubation and IO access. A separate task trainer may be used in parallel for these procedures. |
| --- | --- |
| **Moulage / Appearance** | Clothing: T-shirt, jeans, boxer-briefs, and chest binder (or compressive bandages).  Moulage: Diffuse erythematous, macular rash. Tampon with green/red discharge in vagina.  Equipment: External “pillow” speaker for standardized actor voice or mannequin capable of speech. Single peripheral IV (hidden until requested) connected to a drainage system. |
| **Equipment Available** | Height adjustable gurney  Cardiorespiratory leads  Non-invasive blood pressure cuff  Pulse oximeter  Oxygen delivery supplies: Nasal cannula, simple mask, non-rebreather, BVM with large mask  Standard pediatric code cart  Three-way stopcock  EZ I/O kit: Power driver, 25mm needle set, extension tubing, stabilizer dressing  Intubation equipment: Video laryngoscopy (C-MAC) and direct laryngoscopy laryngoscopes, endotracheal tubes, stylets, and a CO2 detector.  Telephone to call consultants or pharmacist |
| **Medications Available** | Rapid Sequence Intubation Medications: Ketamine, Fentanyl, Midazolam, Rocuronium  Antibiotics: Ceftriaxone, Vancomycin, Piperacillin/Tazobactam, Cefepime, Clindamycin, Linezolid  Fluids: Normal saline, PRBCs, Platelets, Cryoprecipitate, Fresh Frozen Plasma  Vasoactives: Norepinephrine, Epinephrine, Dopamine  Antipyretics: Acetaminophen, Ibuprofen |
