# Supplementary material for: Decompensated Toxic Shock in a Gender-Diverse Adolescent: A Pediatric Emergency Medicine Simulation Case
Source: MedEdPORTAL. 2026 Jul 1;22:11615. doi: 10.15766/mep_2374-8265.11615 (PMC13319108; doi:10.15766/mep_2374-8265.11615)
Supplement: Supplementary file 1 — Simulation Case.docxSimulation Case Equipment.docxStandardized Actor Script.docxCase Materials.pptxDebriefing Outline.docxCritical Actions Checklist.docxPostsimulation Survey.docx [file mep_2374-8265.11615-s001.zip › C. Standardized Actor Script.docx]

**Appendix C: Standardized Actor Script**

*Instructions: This document contains the standardized actor script used to portray the patient during the simulation. The actor should review the script in advance and follow the outlined dialogue, tone, and emotional progression. The actor should respond only to learner prompts and avoid providing unsolicited information unless specified. Responses should reflect the patient’s clinical status and emotional state as the case evolves. The actor should consistently use the patient’s name and pronouns and model appropriate responses to both affirming and non-affirming communication. Dialogue should be delivered in real time via a microphone or in person.*

**Name:** Jessie (they/them)

**Affect:** Nervous, withdrawn

**Background:** You are a 13-year-old presenting to the emergency department for 2 days of abdominal pain and fevers. You started your first period 4 days ago and inserted a tampon to stop the bleeding. However, the tampon became stuck and has remained in place for the past 4 days. You came to the ED on your own because you were embarrassed to tell your family.

**General Guidelines:**

- The patient’s name is “Jessie,” and their pronouns are they/them. The ideal behavior is for the fellow to introduce themselves with their own name and pronouns and ask the patient what their name/pronouns are.
- If the fellow initially uses the wrong name and/or pronouns, you, the standardized patient (SP), should become angry. The SP should become more cooperative as the fellow tries to establish the correct name and pronouns. If the fellow misgenders the SP repeatedly and/or focuses the history-taking on inappropriate questions, the SP should say, “Stop calling me that” or “I don’t use that”
- If the fellow fails to do a GU exam after the first bolus of fluids is given, the SP should say: “My tampon has been stuck for four days, and I can’t get it out”
- During the genitourinary exam, you should adjust your level of anxiety based on how sensitive or gentle the trainee is. For example, if the trainee is abrupt, not talking through all the steps, or not recognizing your baseline anxiety, you should have increased anxiety and impede the trainee’s ability to perform the exam. If, on the other hand, the trainee is calm, professional, gentle, and sensitive, then you should act visibly more relaxed.
- When the facilitator reports that “60 minutes have passed”, you should become progressively more disoriented throughout the remainder of the case.

**Script**

| **Intervention / Time Point** | **Change in Case** | **SP Script** |
| --- | --- | --- |
| 0:00 minutes  Initial Presentation | Learners enter the room to assess the patient. | When the team enters the room: “Who are you?”  Purpose of the visit: “My belly hurts, and I have a fever.”  If the learner asks for more details: “The fevers and belly pain started two days ago. It’s the worst pain I’ve ever had, and I haven’t felt like eating anything.”  If the learner doesn’t address you:  “What’s going on?”  “Can you make my belly pain stop?”  If the learner misgenders you or calls you Jessica (including during communication with other team members), then become angry and say:  “I don’t go by Jessica.”  “You can’t call me she.”  “Stop calling me that.” |
| 0:30 minutes  Initial History | Learner begins to ask about the initial history. | **ROS:**  If asked about fevers: “My temperature was 102.1F yesterday, my whole-body hurts, and I have chills.”  If asked about rashes: “The rash started yesterday. It doesn’t itch. It’s not painful.”  If asked about abdominal pain: “My lower belly hurts, and the pain is getting worse. I started throwing up yesterday and don’t feel like eating anything.”  If asked about pain while urinating: “I don’t have pain when I pee, but there is a smelly green discharge in my underwear.”  If asked about your periods: “I started bleeding for the first time 4 days ago.”  If asked further about their periods: “I tried using a tampon, but I panicked when I put it in and now, I just don’t want to think about it anymore. I don’t know who to go to for help.”  All other systems or diseases are negative.  **Past Medical History:**  “I have no medical problems or surgeries and don't take any medications at home. I usually avoid seeing doctors because they don’t get me, but I will be seeing a specialist next month to hopefully get rid of this bleeding.”    **HEADSS:**  If asked where you live: “I live at home with my parents. I took the bus here alone because I was embarrassed and didn’t want my parents to worry.”  If asked about drugs/alcohol: “I have never tried any drugs or alcohol”  If asked about sexual history: “I have never been sexually active.” |
| 02:00 minutes  Initial Exam | Learner to obtain assent from patient and complete exam. | **Before the GU Exam:**  When told that the learner will need to examine your vagina: “Do you have to?”  If the learner explains the reasoning for the exam: “I really don’t want to, but we can do it if needed.”  If asked if you think learning more about the pelvic exam would be helpful: “Yes, what will you do?”    If asked whether you have any concerns, say, “I’m just nervous that I will freak out or that this will be painful.”  **During the GU Exam:**  Within seconds after the exam starts, you should become very nervous, whispering to yourself “Oh no, Oh no, Oh no” (or something similar). Then say, “I don’t know if I can do this. Is there anything I should be doing?”    If the trainee tries any stress reduction techniques, state that you’re feeling better, and that it’s OK to go ahead with the exam.  If the trainee does not try any stress reduction techniques or acknowledge your discomfort, you should say “I can’t do this. We need to stop this exam.” |
| 04:00 minutes  Initial Work-up and Management | Learner begins work-up / orders OR Bedside RN prompts: “What work-up are you planning to order.” | “What are you planning to do?”  “What do you think is causing my belly pain?” |
| 05:00 minutes  Fluid Refractory Septic/Toxic Shock | Facilitator states: “You continue to see other patients while waiting for labs. Sixty (60) minutes have now gone by.”  Bedside RN: “Can you come back to evaluate this patient? They are tachycardic and do not look well.” | You are slightly more disoriented, requiring prompting twice for some questions, but are still responsive.  If the fellow fails to do a GU exam after the first bolus of fluids is given, say: “My tampon has been stuck for four days, and I can’t get it out” |
| 08:00 minutes  Loss of IV Access, Worsening Mental Status and Respiratory Failure | Pressors are started. | Once pressors are started, you get progressively more disoriented, so that by 1 minute after starting pressors (i.e. when IV access is lost), you are not answering questions and only grunting/moaning to pain. |
