# Supplementary material for: Decompensated Toxic Shock in a Gender-Diverse Adolescent: A Pediatric Emergency Medicine Simulation Case
Source: MedEdPORTAL. 2026 Jul 1;22:11615. doi: 10.15766/mep_2374-8265.11615 (PMC13319108; doi:10.15766/mep_2374-8265.11615)
Supplement: Supplementary file 1 — Simulation Case.docxSimulation Case Equipment.docxStandardized Actor Script.docxCase Materials.pptxDebriefing Outline.docxCritical Actions Checklist.docxPostsimulation Survey.docx [file mep_2374-8265.11615-s001.zip › D. Case Materials.pptx]

## Slide 1
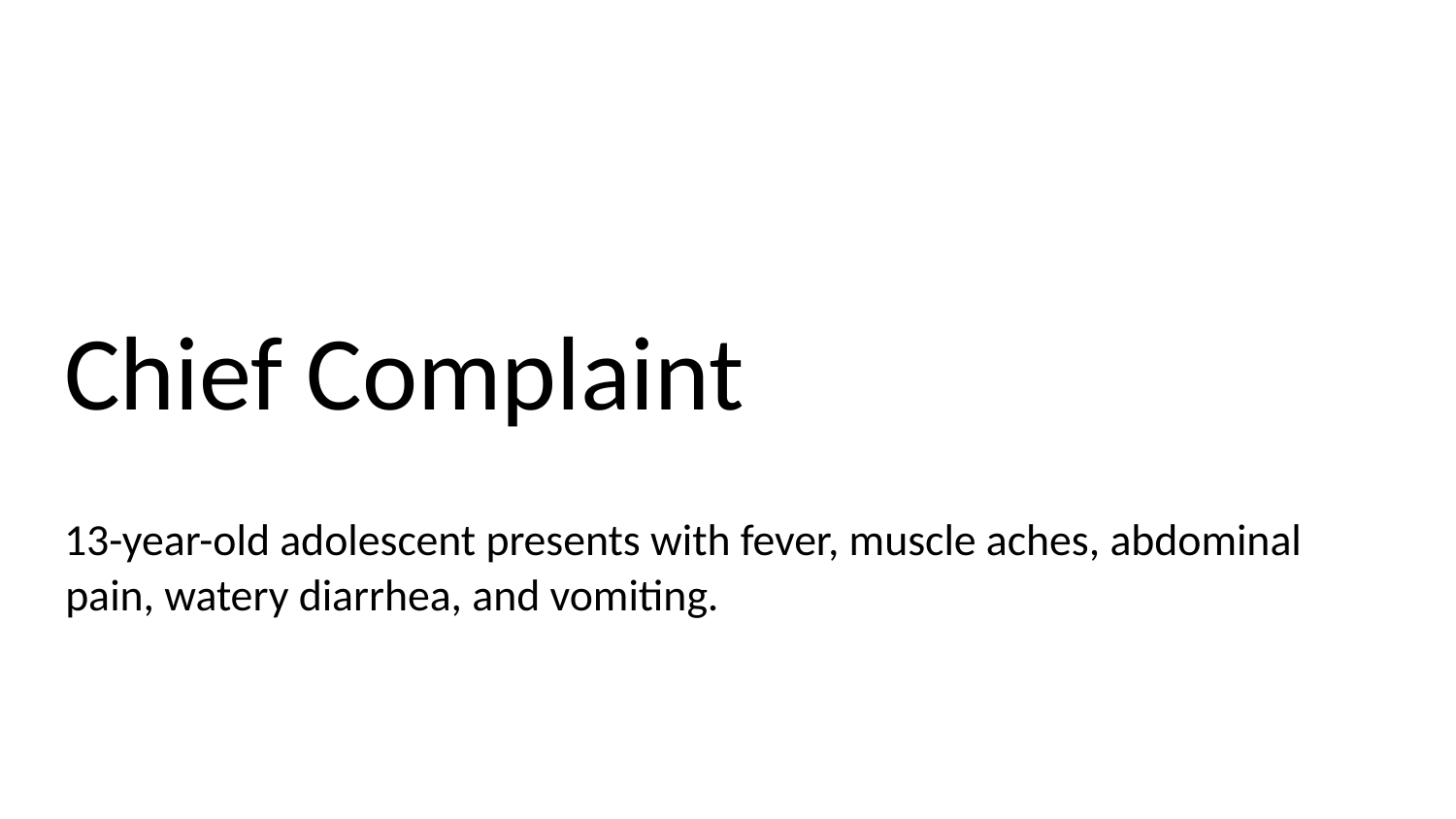

# Chief Complaint
13-year-old adolescent presents with fever, muscle aches, abdominal pain, watery diarrhea, and vomiting.

## Slide 2
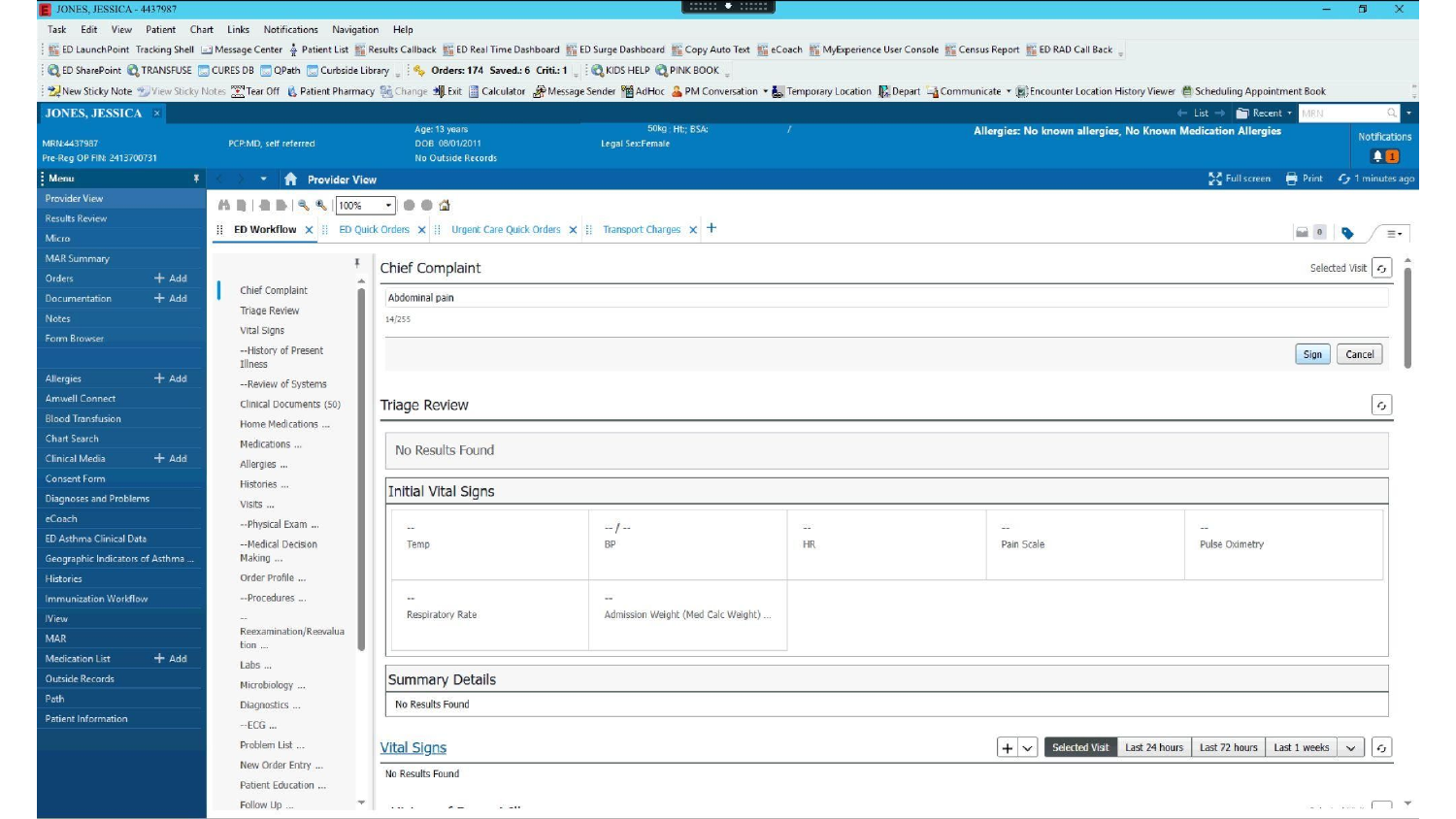

## Slide 3
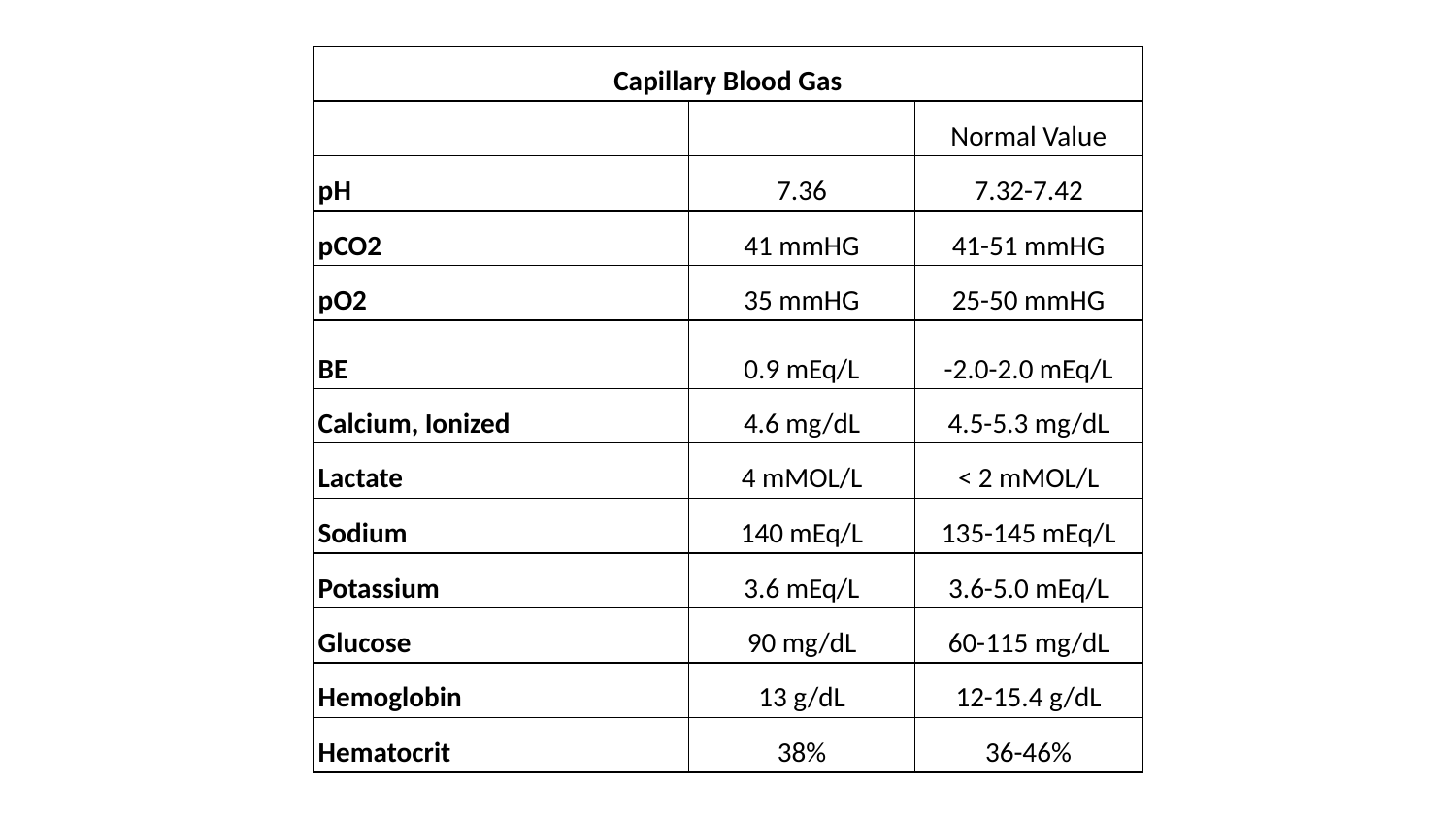

| Capillary Blood Gas | | |
| --- | --- | --- |
| | | Normal Value |
| pH | 7.36 | 7.32-7.42 |
| pCO2 | 41 mmHG | 41-51 mmHG |
| pO2 | 35 mmHG | 25-50 mmHG |
| BE | 0.9 mEq/L | -2.0-2.0 mEq/L |
| Calcium, Ionized | 4.6 mg/dL | 4.5-5.3 mg/dL |
| Lactate | 4 mMOL/L | < 2 mMOL/L |
| Sodium | 140 mEq/L | 135-145 mEq/L |
| Potassium | 3.6 mEq/L | 3.6-5.0 mEq/L |
| Glucose | 90 mg/dL | 60-115 mg/dL |
| Hemoglobin | 13 g/dL | 12-15.4 g/dL |
| Hematocrit | 38% | 36-46% |

## Slide 4
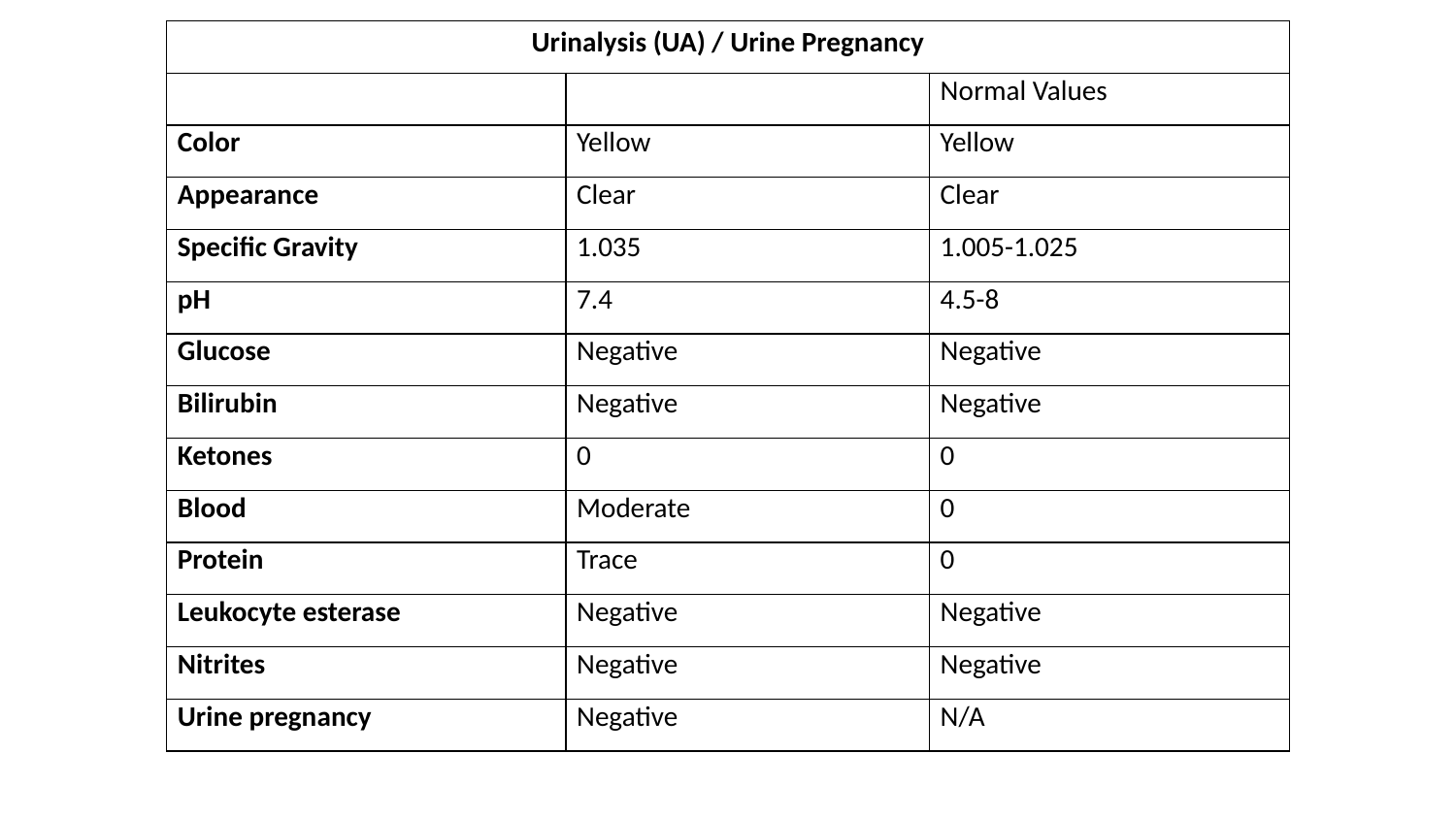

| Urinalysis (UA) / Urine Pregnancy | | |
| --- | --- | --- |
| | | Normal Values |
| Color | Yellow | Yellow |
| Appearance | Clear | Clear |
| Specific Gravity | 1.035 | 1.005-1.025 |
| pH | 7.4 | 4.5-8 |
| Glucose | Negative | Negative |
| Bilirubin | Negative | Negative |
| Ketones | 0 | 0 |
| Blood | Moderate | 0 |
| Protein | Trace | 0 |
| Leukocyte esterase | Negative | Negative |
| Nitrites | Negative | Negative |
| Urine pregnancy | Negative | N/A |

## Slide 5
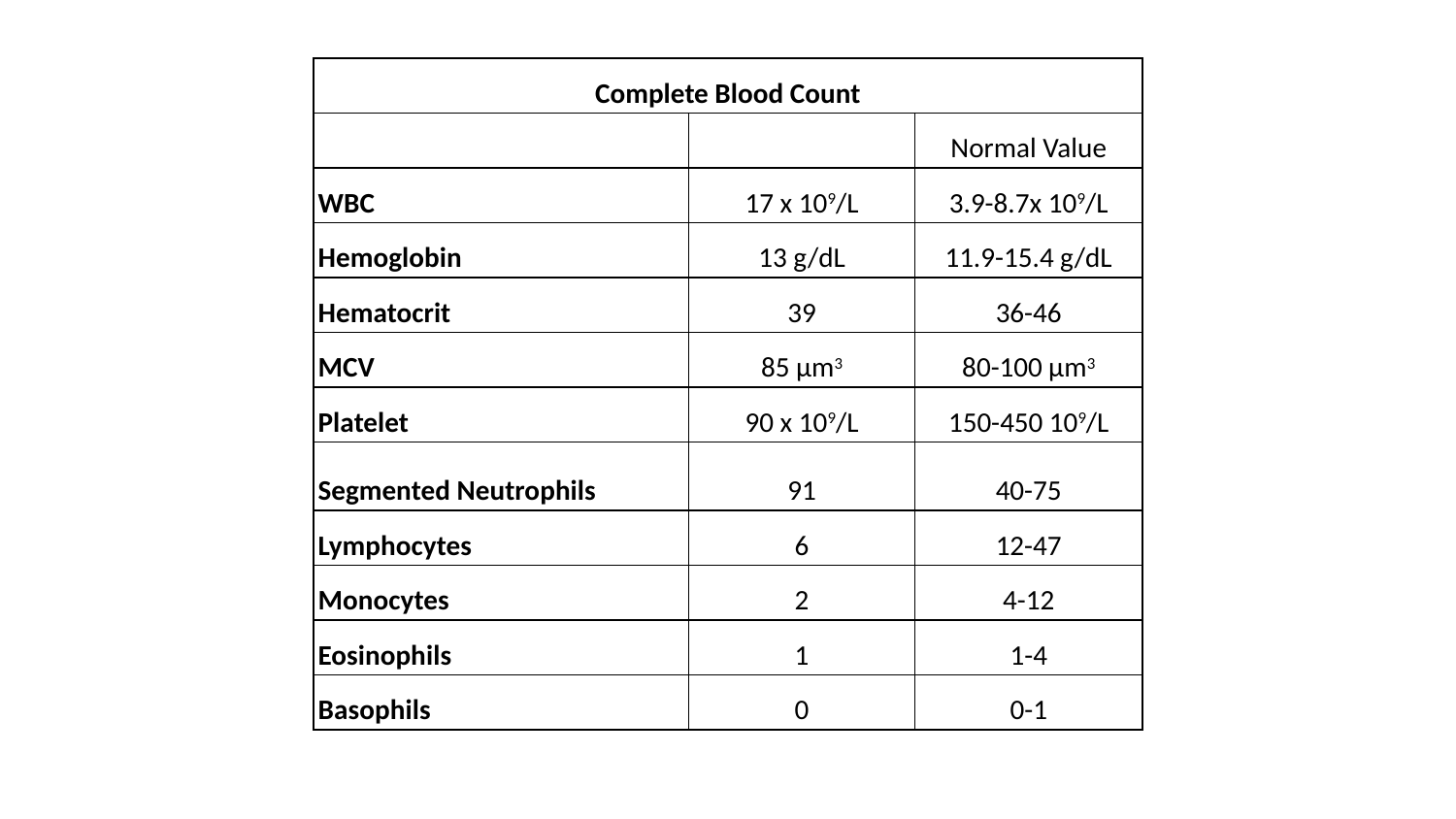

| Complete Blood Count | | |
| --- | --- | --- |
| | | Normal Value |
| WBC | 17 x 109/L | 3.9-8.7x 10^9^/L |
| Hemoglobin | 13 g/dL | 11.9-15.4 g/dL |
| Hematocrit | 39 | 36-46 |
| MCV | 85 µm3 | 80-100 µm3 |
| Platelet | 90 x 109/L | 150-450 109/L |
| Segmented Neutrophils | 91 | 40-75 |
| Lymphocytes | 6 | 12-47 |
| Monocytes | 2 | 4-12 |
| Eosinophils | 1 | 1-4 |
| Basophils | 0 | 0-1 |

## Slide 6
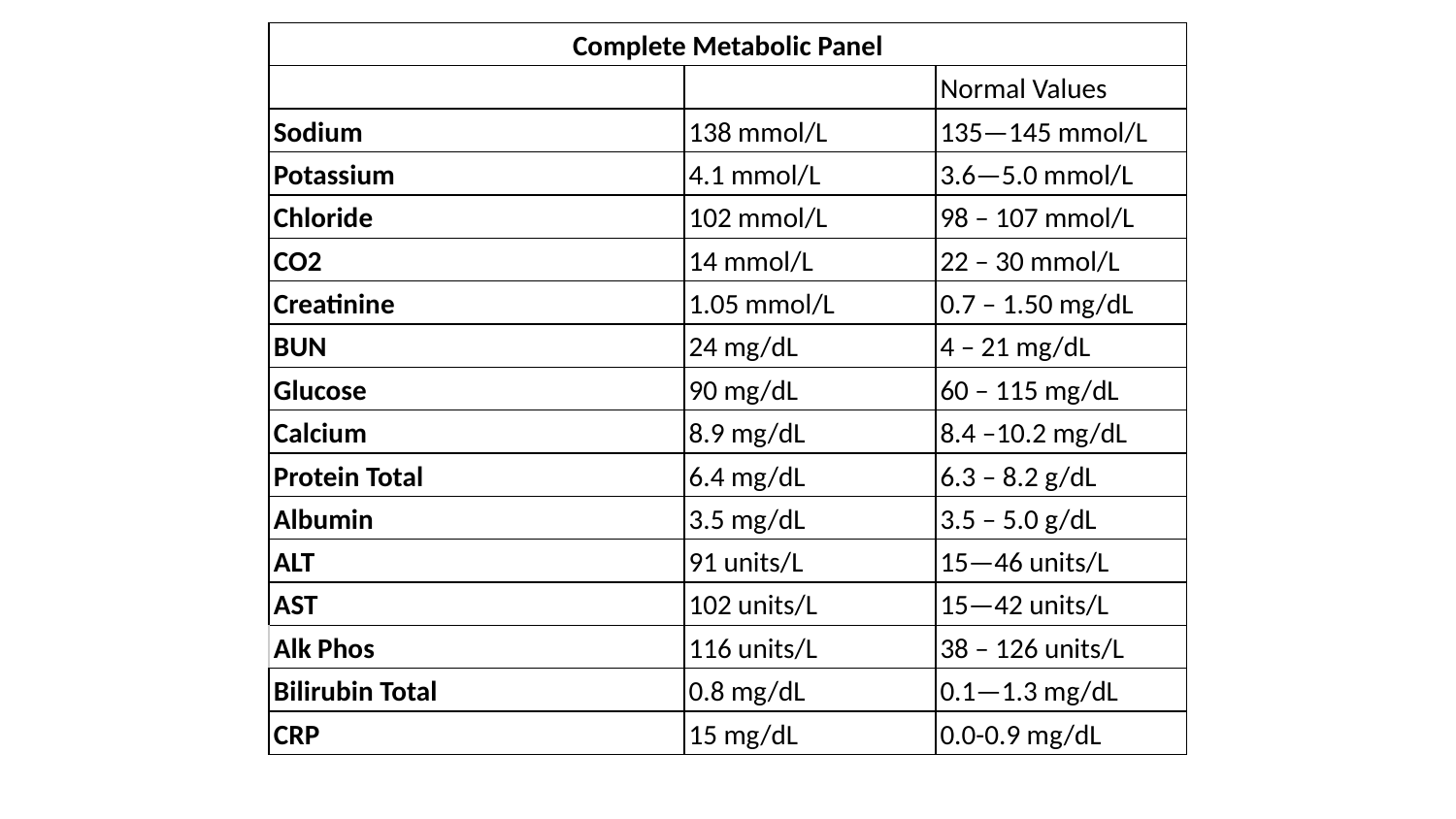

| Complete Metabolic Panel | | |
| --- | --- | --- |
| | | Normal Values |
| Sodium | 138 mmol/L | 135—145 mmol/L |
| Potassium | 4.1 mmol/L | 3.6—5.0 mmol/L |
| Chloride | 102 mmol/L | 98 – 107 mmol/L |
| CO2​​ | 14 mmol/L | 22 – 30 mmol/L |
| Creatinine | 1.05 mmol/L | 0.7 – 1.50 mg/dL |
| BUN | 24 mg/dL | 4 – 21 mg/dL |
| Glucose | 90 mg/dL | 60 – 115 mg/dL |
| Calcium | 8.9 mg/dL | 8.4 –10.2 mg/dL |
| Protein Total | 6.4 mg/dL | 6.3 – 8.2 g/dL |
| Albumin | 3.5 mg/dL | 3.5 – 5.0 g/dL |
| ALT | 91 units/L | 15—46 units/L |
| AST | 102 units/L | 15—42 units/L |
| Alk Phos | 116 units/L | 38 – 126 units/L |
| Bilirubin Total | 0.8 mg/dL | 0.1—1.3 mg/dL |
| CRP | 15 mg/dL | 0.0-0.9 mg/dL |

## Slide 7
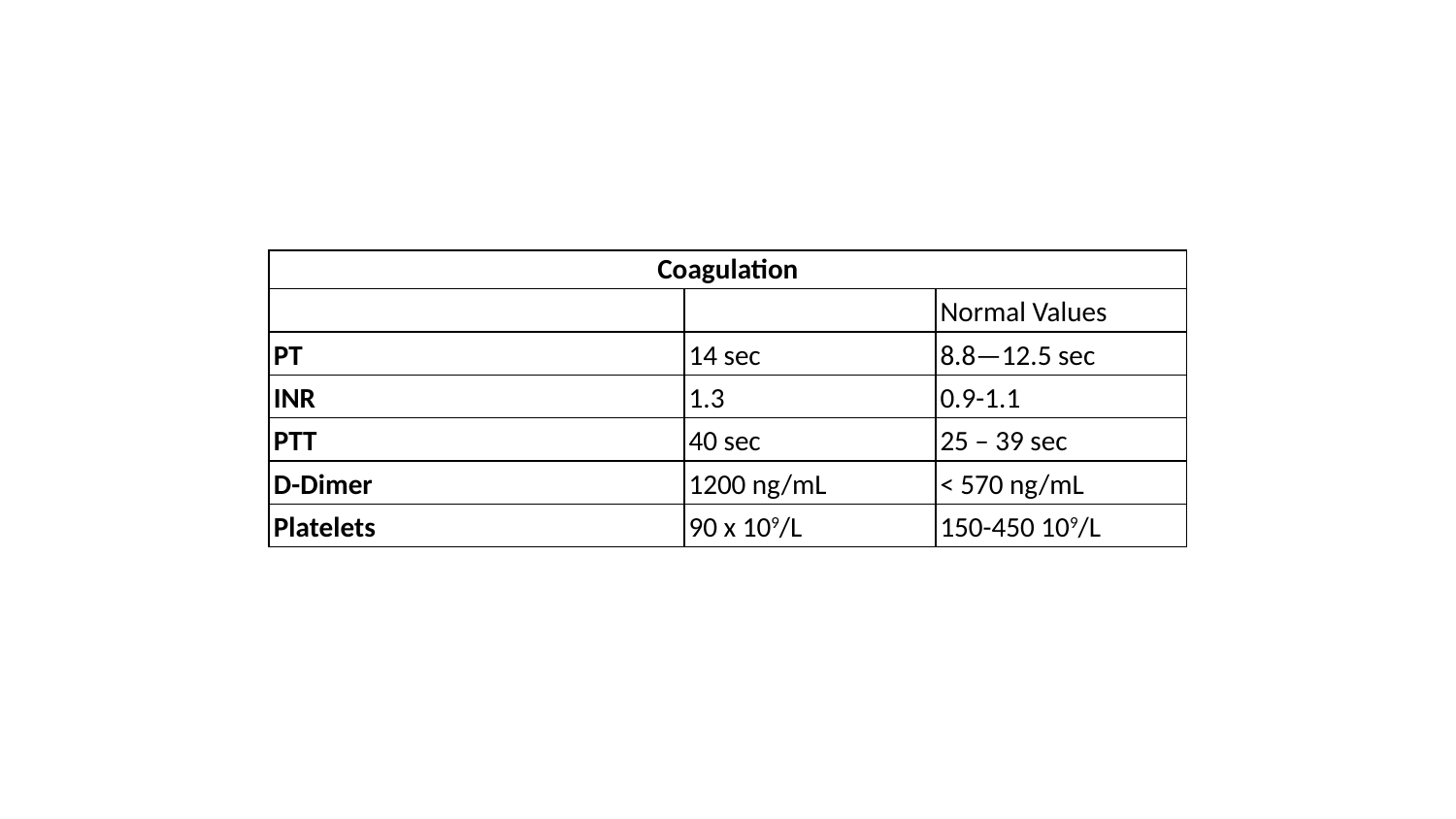

| Coagulation | | |
| --- | --- | --- |
| | | Normal Values |
| PT | 14 sec | 8.8—12.5 sec |
| INR | 1.3 | 0.9-1.1 |
| PTT | 40 sec | 25 – 39 sec |
| D-Dimer | 1200 ng/mL | < 570 ng/mL |
| Platelets | 90 x 109/L | 150-450 109/L |

## Slide 8
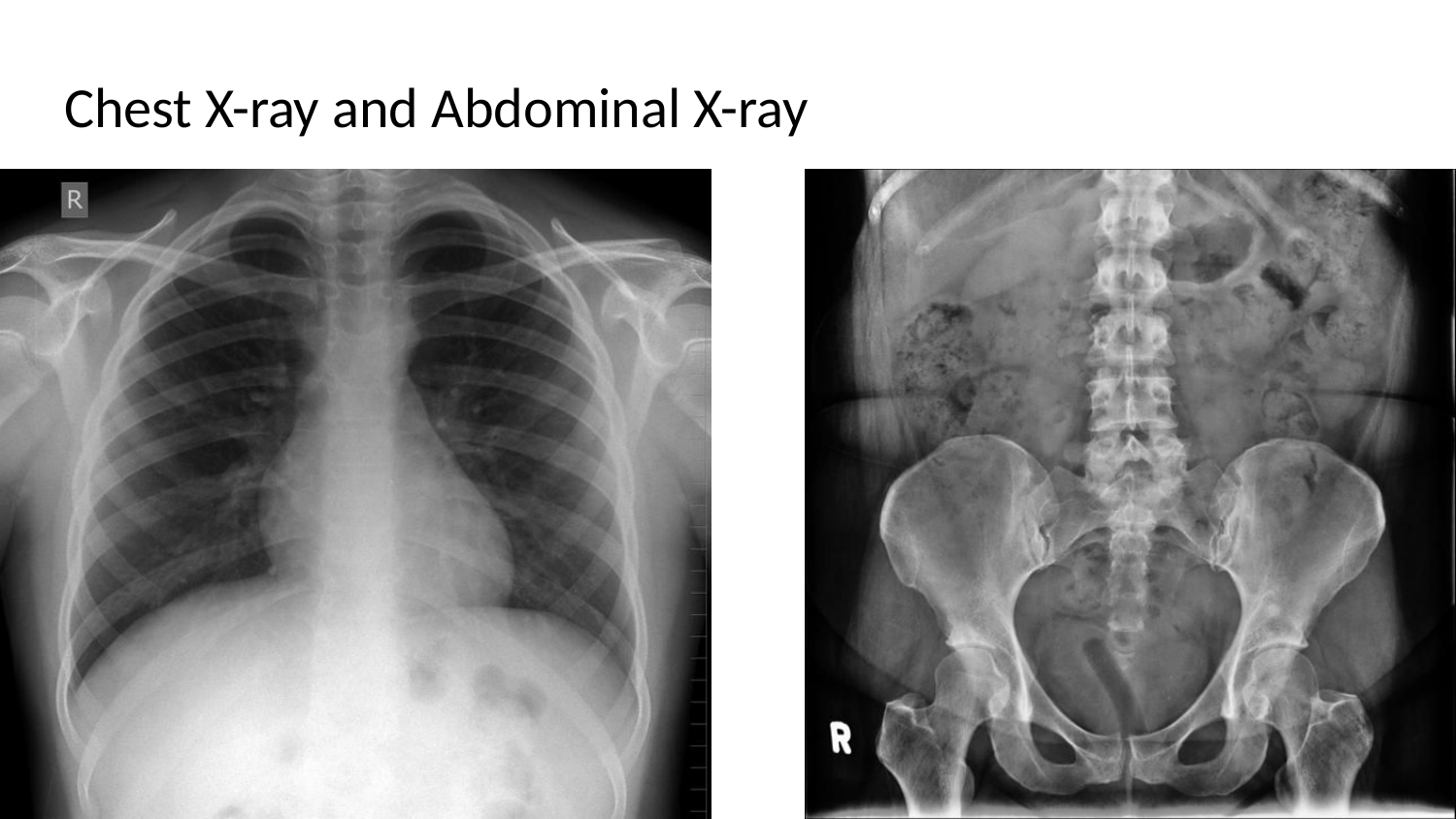

# Chest X-ray and Abdominal X-ray

## Slide 9
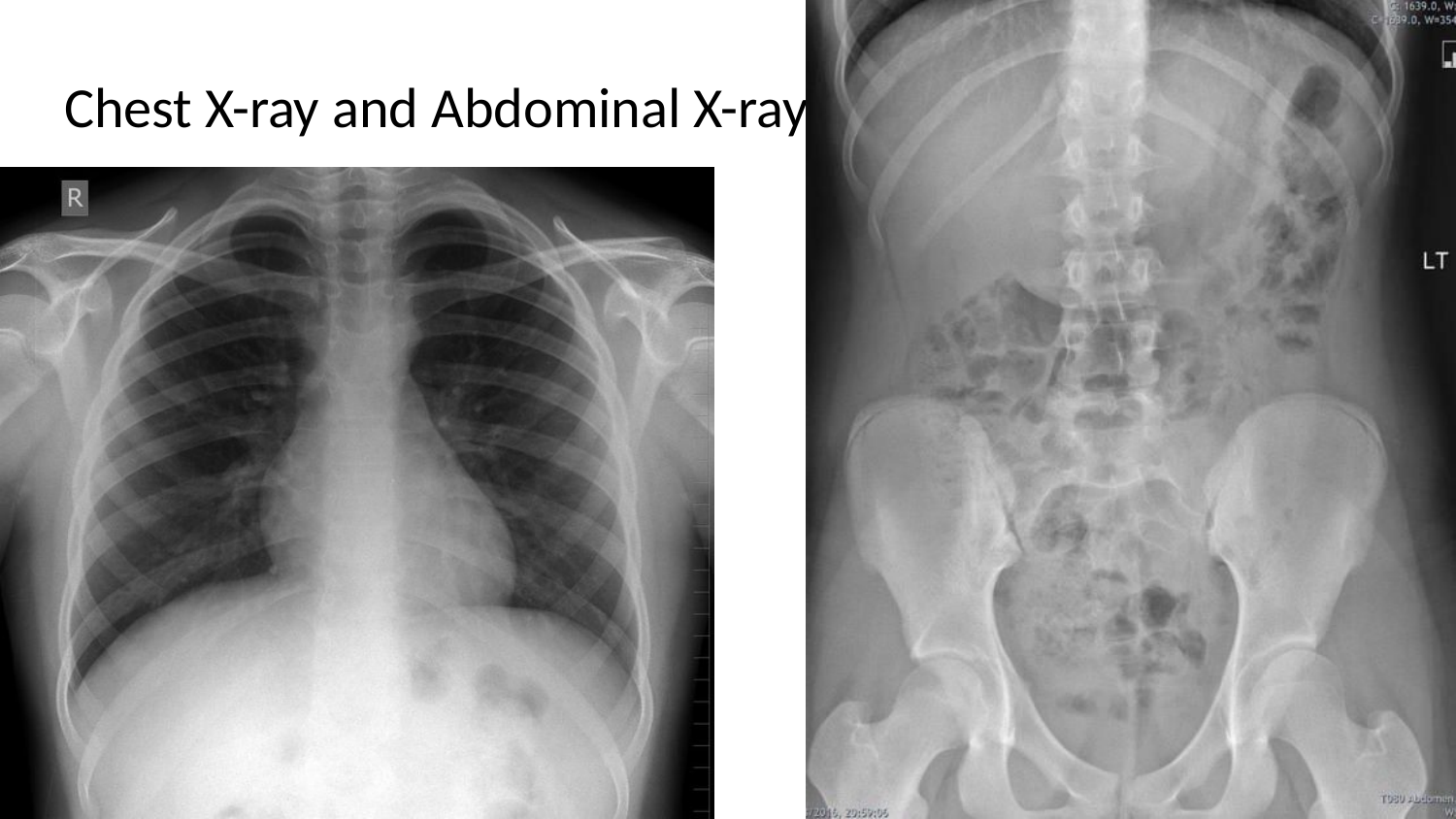

# Chest X-ray and Abdominal X-ray

## Slide 10
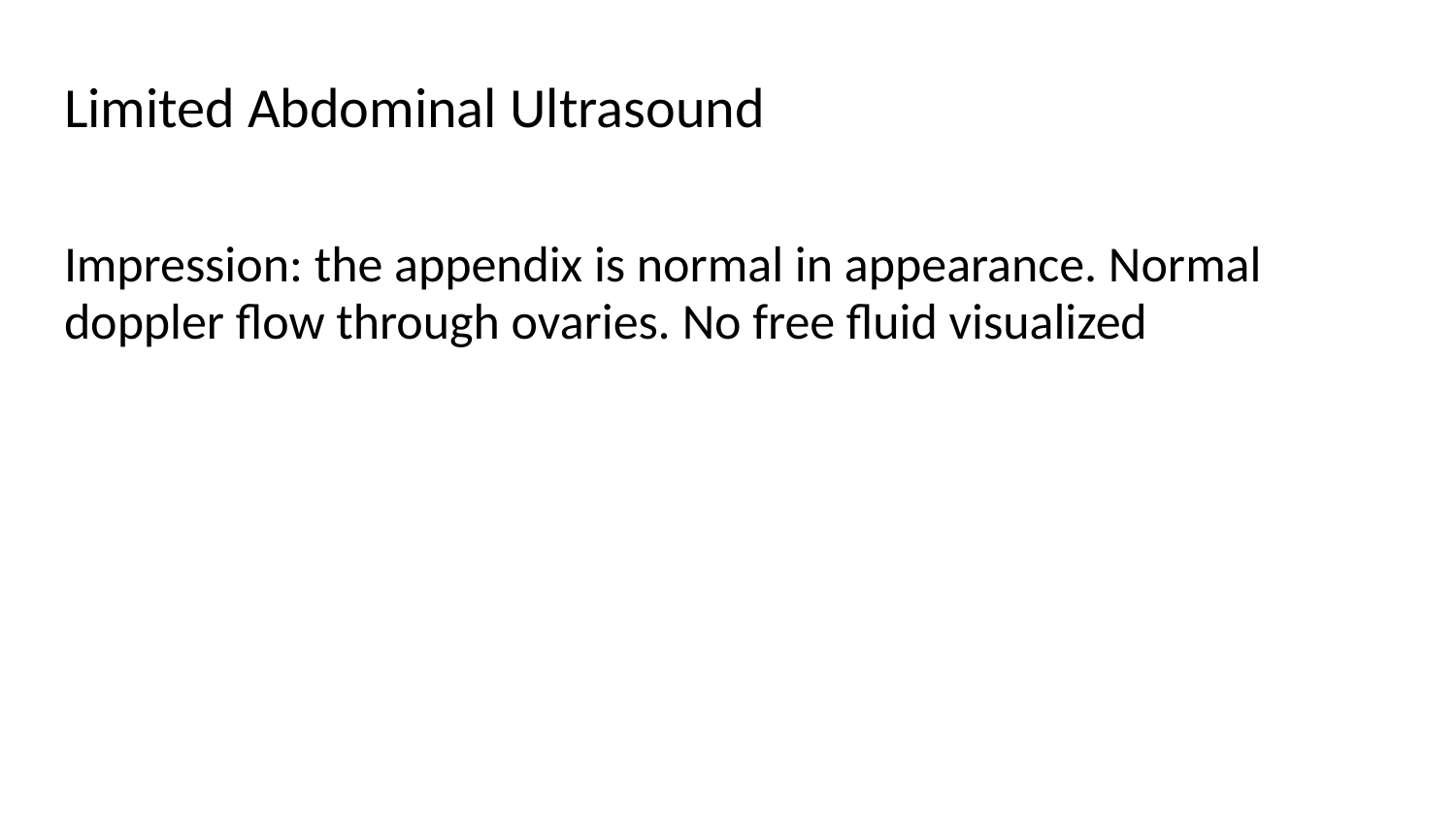

# Limited Abdominal Ultrasound
Impression: the appendix is normal in appearance. Normal doppler flow through ovaries. No free fluid visualized

## Slide 11
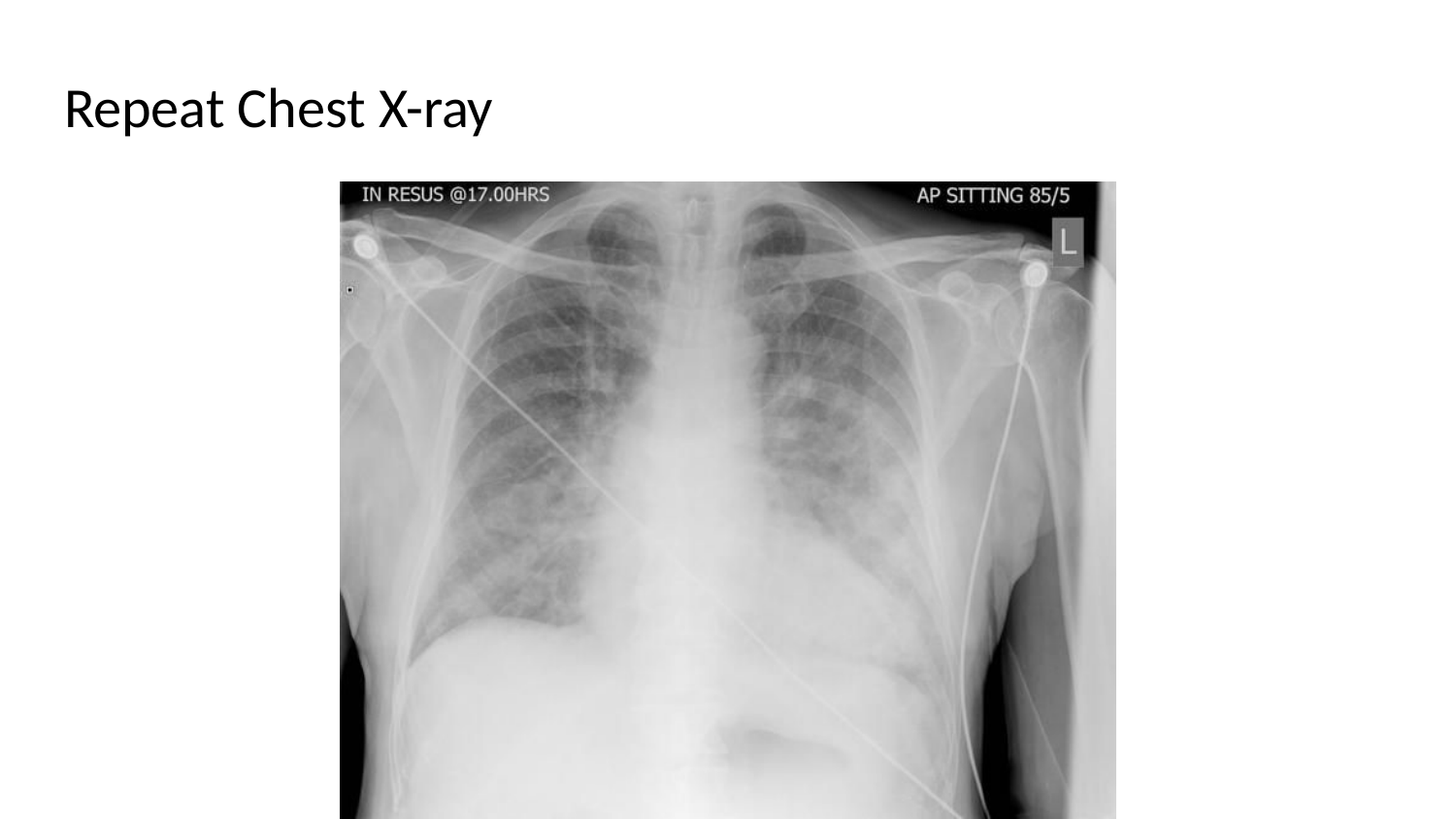

# Repeat Chest X-ray
