# Supplementary material for: Decompensated Toxic Shock in a Gender-Diverse Adolescent: A Pediatric Emergency Medicine Simulation Case
Source: MedEdPORTAL. 2026 Jul 1;22:11615. doi: 10.15766/mep_2374-8265.11615 (PMC13319108; doi:10.15766/mep_2374-8265.11615)
Supplement: Supplementary file 1 — Simulation Case.docxSimulation Case Equipment.docxStandardized Actor Script.docxCase Materials.pptxDebriefing Outline.docxCritical Actions Checklist.docxPostsimulation Survey.docx [file mep_2374-8265.11615-s001.zip › E. Debriefing Outline.docx]

**Appendix E: Debriefing Outline**

*Instructions: This document provides a structured debriefing guide based on the PEARLS framework^1^. Facilitators should conduct the debrief immediately after the simulation. The guide includes suggested prompts aligned with the learning objectives, addressing clinical management, communication, and trauma-informed care. Facilitators may* *adapt questions based on learner performance while ensuring that all key objectives are discussed. The recommended debriefing duration is approximately 30 minutes.*

**Setting the Scene:**

*We are now going to take the next 20 minutes to debrief. Our goal is to improve how we work together and care for our patients. To create a safe space for open dialogue, we will be following the Safer Spaces Framework.*

SAFER Spaces Framework^2^

- Sharing openly, listening to understand, and maintaining confidentiality.
- Assuming positive intent and holding space for yourself and others.
- Fostering growth and reflective practice with flexibility and mindfulness.
- Expecting and embracing discomfort.
- Respecting each other’s experiences with humility and empathy for yourself and others.

**Reactions:**

*That was a challenging case.* *Let’s talk about how things went. Any initial reactions? How are you feeling?*

**Description:**

*Can someone please share a summary of the case?*

**Analysis:**

1. Gender Identity/Pronouns^3^

*How did the patient’s identity differ from what was listed in the EMR?*

*What strategies could you use going forward to ensure that you treat transgender patients with respect, given the constraints of electronic-based records?*

*What are your thoughts regarding why the patient presented alone to the ED?*

*Do you think the patient was delayed in their presentation? What might be the motivating factors for the delay?*

*Why do you think Jessie had issues with tampon use?*

- Introduce yourself using your name and pronouns and ask each patient for their name and pronouns. For example, ask “What name do you go by, and what are your pronouns?” In high-intensity situations, you can opt to use gender-neutral language when you are unsure of a patient’s pronouns.
- Always use the correct name and pronouns of patients, even when they are not present and create an environment of accountability for others. This establishes a safe environment in which a patient can feel comfortable advocating for themselves and asserting their pronouns. If incorrect pronouns are used, this can lead to patients feeling alienated and invalidated. They may not feel safe seeking care for future health issues.
- We will make mistakes. Apologize and try to use names/pronouns correctly. It is okay not to know**,** but it is not okay to not be open to learning. For example, say “I’m sorry for using the wrong pronoun. What are your pronouns?” or “I apologize. I’m still learning. Let me try again.”
- Avoid asking unnecessary questions.
- Review how to update pronouns in the electronic medical record.
- Because the patient presented alone to the ED, it is possible that they may not be out to their family members and have concerns about protecting their gender identity. This may also be a reason the patient was delayed in their presentation. If appropriate, ask the patient what their comfort level is with their family. Ask what pronouns and names they would like to use with and without family present. As with all adolescents, it is important to have time/space for private discussions to establish an environment of confidentiality. Consider visibility of the EMR and options such as making a confidential note to document these discussions.
- Tampon use can exacerbate gender dysphoria, which may result in increased psychological stress or fear when discussing the tampon with the patient, especially because this patient is experiencing their first period.
- Upon discharge, consider offering options for the patient to seek care in the future, including APLA Health, Planned Parenthood, Folx Health, or other local options. This is an evolving landscape with many barriers to care depending on where a patient lives.

2. Trauma-Informed Care

*How did the physical exam portion of the exam go?*

*How did you feel seeing the patient distressed by the idea of having to have a physical exam, yet knowing you had to do it for their safety?*

*How can we conduct quick assessments or recognize emotional reactions to crises/potentially traumatic events?*

*How can we react in the moment by taking a trauma-informed approach?*

- D-E-F Framework^4^
  - Address Distress: optimize pain management and help children cope with fears or worries
    - Provide the child with as much control as possible over the clinical encounter. The child should understand what is about to happen and have a say in what is about to happen (procedural positioning). Ask about fears and worries and provide simple explanations for medical procedures to reduce anxiety. After explaining the diagnosis or procedure, ask the child to say it to you to hear how the child understands what is happening. Clarify misconceptions by providing accurate information and using words and ideas the child can understand.
    - Acknowledge common emotional reactions to trauma and traumatic stress. Provide reassurance and realistic hope by describing what is being done to help the child get better and stating that many people are working together to help the child.
  - Facilitate Emotional Support: assess the child’s need for emotional support and then help the health care team provide support effectively
    - Engage the child in active distraction before and during a procedure such as involving them in non-procedural conversations about things they enjoy, offering a squeeze ball or listening to preferred music.
    - When a child becomes distressed, provide specific instructions rather than reassurance. For example, say “breathe in through your nose as deep as you can.” Research has found that verbal reassurance (“You’re OK” or “Don’t worry”) can exacerbate a child’s pain and distress. Remember to praise positive behavior.
  - Remember the Family: identify needs, strengths, and resources
    - Not applicable to the simulation case as family was not present for the encounter.
- Specific guidance for the pelvic exam^5^
  - Address pain by offering a benzodiazepine 20 minutes before the exam for those with severe anxiety. Before starting, remind the patient that the exam can be stopped anytime at their request. Explain each step in a clear and direct way. For example, say “I will touch with my hand now,” “you will experience some pressure next” or “you will hear the clicking noise of the speculum now.” Avoid using medical terms for body parts unless discussed beforehand that these are preferred terms the patient would like you to use. Some patients may prefer the terms “front” or “front hole” rather than “vagina.” Apply stress reduction techniques such as deep breathing, mental grounding techniques (orient the patient to the location, date) or physical ground techniques (make a fist and squeeze, hold the table).

3. Toxic Shock^6,7,8,9,10^

*What did you determine to be the patient’s primary presenting diagnosis and why?*

*How did you choose to approach the management of the patient once a diagnosis was established?*

*Reflecting back, what changes would you have made in your management and why?*

- Recognition
  - Pathogenesis: Toxic shock is a type of septic shock with a key difference. Symptoms from toxic shock are due to release of bacterial exotoxin produced by Staph aureus or group A strep, which act on T-cells to cause massive cytokine release, as compared to septic shock where symptoms are due to dysregulation of immune response.
  - History/Exam: Patients present with sudden onset of high fever, vomiting, and watery diarrhea. There are a lot of crossovers with the presenting symptoms of septic shock including fever or hypothermia, tachycardia, hypotension, delayed or flash capillary refill, but certain clues may suggest presence of toxic shock. Patients with toxic shock have a diffuse, erythematous, maculopapular rash and classically a history of recent surgery, menses or retained foreign bodies. However, in almost one-half of cases, no portal of entry is identified.
  - Laboratory findings: Serum studies are notable for leukocytosis with left shift, thrombocytopenia, transaminitis, elevated creatinine, elevated creatine kinase, myoglobinuria, and coagulopathy.
- Management: The management of toxic shock is similar but not the same as septic shock. Both include fluid resuscitation, antibiotics, and potentially vasoactive medications. However, toxic shock also requires an additional antibiotic that blocks protein synthesis and removal of the nidus of infection when possible.
  - Fluids: Patients should receive IV fluid resuscitation with crystalloids using a push-pull technique, pressure bag, or rapid infuser as needed. Start with 20mL/kg and continue to give additional fluid boluses up to 40-60mL/kg, titrating to goal clinical parameters (urine output, improved perfusion, improved heart rate, or blood pressure). Keep in mind that the amount of fluid resuscitation differs between high resource and low resource settings. Evaluate the patient between each bolus to monitor for fluid overload.
  - Antibiotics: Broad-spectrum antibiotics (e.g., ceftriaxone +/- vancomycin) should be administered to patients with an unidentified organism. Clindamycin or linezolid should be added to suppress toxin production.
  - Vasoactives: Vasopressors (epinephrine or norepinephrine) should be administered for toxic shock refractory to 40-60mL/kg of fluid resuscitation or sooner if signs of fluid overload or myocardial dysfunction are present.
  - Oxygenation/Ventilation: Maintain SpO2 over 90% with supplemental oxygen support if needed. Assess gas exchange using clinical assessment (respiratory rate, work of breathing, capnography, breath sounds) and blood gas. Consider non-invasive positive pressure ventilation (HFNC, BiPAP, CPAP) to assist with ventilation if the patient has increased work of breathing or inadequate gas exchange on the blood gas (elevated pCO2). Consider intubation for hypoxemia, refractory shock, or declining mental status to assist with ventilation, protect airway, and/or promote oxygenation.

4. Pulmonary Edema and Respiratory Failure^9,10^

*What decisions were made regarding airway management? What prompted these decisions / why were these decisions made?*

*Did you recognize that the patient developed pulmonary edema? How was this recognized? If not, what do you think prevented the team from recognizing the presence of pulmonary edema?*

- Signs and symptoms: During fluid resuscitation, worsening tachypnea, hypoxemia, cough, or new/worsening crackles suggests pulmonary edema. These findings can be confirmed by the presence of new opacities on CXR.
- Management: It is important to frequently reassess patients after each fluid bolus. If pulmonary edema is recognized or the patient is deemed to be in fluid non-responsive shock, start vasoactive medications and stop rapid fluid resuscitation. During sepsis, avoid diuresis in the initial phase as increasing intravascular volume should be the priority.

5. IO Placement^11^

*What was your thought process when you realized that the patient had lost IV access?*

*How did the placement of the IO needle go?*

- Overview: Intraosseous (IO) infusion is indicated when emergent IV access is required, but intravenous access cannot be rapidly obtained. IO access can be obtained in an awake patient. Contraindications to IO access include a fracture or prior IO attempt at the site or if there is an overlying cellulitis or burn (relative contraindication. The functional capabilities of IO are listed below:
  - Labs:
    - Good: H/H, glucose, BUN/Cr, and chloride
    - Relative: Sodium (within 5%), Calcium (within 10%)
    - Poor: WBC (generally higher than true value), Plt (usually lower than true value), potassium (usually hemolyzed), CO2
    - Do not run on blood gas machine
  - Medications:
    - Almost all medications (including antibiotics, vasoactives, fluids, blood products) can be given. Not recommended for adenosine due to short half-life of medication.
    - Optimal flow rates obtained via administration under pressure
- Procedure
  1. Collect supplies including EZ-IO kit [Arros/Teleflex]. Assemble the EZ-IO device.
  2. Identify and clean insertion site with antiseptic solution.
     1. Preferred site is proximal tibia at the flat, medial surface 1-3 cm inferior to tibial tuberosity.
  3. Consider local analgesia with 1% or 2% lidocaine (skin and periosteum). Do not delay insertion in critically ill patients.
  4. Place the IO needle, perpendicularly, into the skin at the insertion site until bone is felt. At least one black line should be visible above skin.
  5. Apply constant pressure perpendicular to the bone while constantly squeezing EZ-IO trigger until loss of resistance if felt.
  6. Unscrew and remove the trocar.
  7. Apply EZ-IO stabilizer.
  8. May attempt to aspirate marrow to confirm placement (not required).
  9. Attach and flush tubing. If the patient is awake, consider an infusion of 2% lidocaine over 1 minute to help with pain.

**Application/Summary:**

*Thank you for this rich discussion. What are some takeaways from this discussion for our clinical practice?*

**References**

1. Bajaj K, Meguerdichian M, Thoma B, Huang S, Eppich W, Cheng A. The PEARLS Healthcare Debriefing Tool. Acad Med. 2018, 93(2), 336.
2. CHLA ACGME Teaching and Learning Collaborative Curriculum Workgroup on Cultural Humility. SAFER Spaces Framework.
3. Keuroghlian AS. “Sensitive and Affirming Communication” from the Harvard Medical School Sexual and Gender Minority Health Faculty Development Module Series. 2022.

### The D-E-F Framework for Trauma-Informed Pediatric Care. Healthcare Toolbox. <https://www.healthcaretoolbox.org/d-e-f-framework-trauma-informed-care>. Accessed December 28, 2024.

1. Guidelines for the Primary and Gender-Affirming Care of Transgender and Gender Nonbinary People. University of California San Francisco Gender Affirming Health Program. Updated June 17, 2016. Accessed October 6, 2024. [https://transcare.ucsf.edu/guidelines](https://transcare.ucsf.edu/guidelines/physical-examination)
2. Ross A, Shoff HW. Toxic Shock Syndrome. In: *StatPearls*. Treasure Island (FL): StatPearls Publishing; August 7, 2023.
3. Evans L, Rhodes A, Alhazzani W, et al. Surviving Sepsis Campaign: International Guidelines for Management of Sepsis and Septic Shock 2021. *Crit Care Med*. 2021;49(11):e1063-e1143. doi:10.1097/CCM.0000000000005337
4. Maitland K, Kiguli S, Opoka RO, et al. Mortality after fluid bolus in African children with severe infection. *N* *Engl J Med*. 2011;364(26):2483-2495. doi:10.1056/NEJMoa1101549
5. Lasa JJ, Dhillon GS, Duff JP, et al. Part 8: Pediatric Advanced Life Support: 2025 American Heart Association and American Academy of Pediatrics Guidelines for Cardiopulmonary Resuscitation and Emergency Cardiovascular Care. *Circulation*. 2025;152(16_suppl_2):S479-S537. doi:10.1161/CIR.0000000000001368
6. Shaw KN, Bachur RG, Chamberlain JM, et al. *Fleisher & Ludwig's Textbook of Pediatric Emergency Medicine,* *8e*. Lippincott Williams & Wilkins, a Wolters Kluwer business; 2021. Accessed November 06, 2025. https://emergency.lwwhealthlibrary.com/book.aspx?bookid=2984&sectionid=0
7. Teleflex Incorporated. *Arrow EZ-IO Intraosseous Vascular Access System: Pocket Guide.* MCI-100779-EN-ANZ, Rev 0. March 2022. Teleflex; 2022. Accessed November 10, 2025. Available from:<https://www.teleflex.com/ezioeducation>
