# Supplementary material for: Decompensated Toxic Shock in a Gender-Diverse Adolescent: A Pediatric Emergency Medicine Simulation Case
Source: MedEdPORTAL. 2026 Jul 1;22:11615. doi: 10.15766/mep_2374-8265.11615 (PMC13319108; doi:10.15766/mep_2374-8265.11615)
Supplement: Supplementary file 1 — Simulation Case.docxSimulation Case Equipment.docxStandardized Actor Script.docxCase Materials.pptxDebriefing Outline.docxCritical Actions Checklist.docxPostsimulation Survey.docx [file mep_2374-8265.11615-s001.zip › F. Critical Actions Checklist.docx]

**Appendix F: Critical Actions Checklist**

*Instructions: This document contains a critical* *actions checklist to assess learner performance. This checklist should be printed or available electronically for the facilitator to complete in real time during the simulation. Items correspond to key learning objectives and are intended to capture observable learner behaviors.*

| **Objective** | **Completed** |
| --- | --- |
| 1. Utilized gender-neutral language until asking for and confirming the patient’s name and pronouns. |  |
| 2. Established and consistently used the patient’s correct name and pronouns throughout the case. |  |
| 3. Performed a trauma-informed pelvic exam using the patient’s preferred anatomical terminology. |  |
| 4. Verbalized recognition of toxic shock signs: fever, rash, tachycardia, hypotension, and delayed capillary refill. |  |
| 5. Administered appropriate antibiotics (including those that inhibit protein synthesis) within one minute of recognizing toxic shock. |  |
| 6. Delivered at least 20 mL/kg (up to 60 mL/kg) of isotonic fluids promptly using push-pull technique. |  |
| 7. Recognized signs of pulmonary edema. |  |
| 8. Stopped intravenous fluids and initiated vasopressors when patient develops pulmonary edema. |  |
| 9. Obtained intraosseous access after failure to establish peripheral IV access. |  |
| 10. Intubated the patient after signs of poor mental status (GCS < 8) and respiratory failure. |  |
