# Supplementary material for: Decompensated Toxic Shock in a Gender-Diverse Adolescent: A Pediatric Emergency Medicine Simulation Case
Source: MedEdPORTAL. 2026 Jul 1;22:11615. doi: 10.15766/mep_2374-8265.11615 (PMC13319108; doi:10.15766/mep_2374-8265.11615)
Supplement: Supplementary file 1 — Simulation Case.docxSimulation Case Equipment.docxStandardized Actor Script.docxCase Materials.pptxDebriefing Outline.docxCritical Actions Checklist.docxPostsimulation Survey.docx [file mep_2374-8265.11615-s001.zip › G. Postsimulation Survey.docx]

**Appendix G: Postsimulation Survey**

*Instructions: This document contains a post-simulation survey to assess learners’ self-reported knowledge, confidence, and perceptions of the learning objectives. The survey should be distributed immediately following the debrief. In the pilot implementation, learners accessed the survey via a QR code linked to Microsoft Forms; alternative distribution methods may be used based on local resources. Participation should be voluntary and anonymous.*

1. Prior to today, my medical training so far had prepared me to effectively provide medical care for transgender patients.
   1. Strongly agree
   2. Agree
   3. Neither disagree nor agree
   4. Disagree
   5. Strongly disagree
2. The experience today increased my knowledge and skills to effectively provide medical care for transgender patients.
   1. Strongly agree
   2. Agree
   3. Neither disagree nor agree
   4. Disagree
   5. Strongly disagree
3. How effective do you believe this simulation-based exercise was in preparing you to interact with patients using trauma-informed care principles?
   1. Extremely effective
   2. Very effective
   3. Somewhat effective
   4. Slightly effective
   5. Not at all effective
4. The simulation case was effective in teaching recognition of toxic shock.
   1. Strongly agree
   2. Agree
   3. Neither disagree nor agree
   4. Disagree
   5. Strongly disagree
5. The simulation case better prepared me to stabilize a patient with toxic shock.
   1. Strongly agree
   2. Agree
   3. Neither disagree nor agree
   4. Disagree
   5. Strongly disagree
6. The simulation case was effective in teaching management of toxic shock.
   1. Strongly agree
   2. Agree
   3. Neither disagree nor agree
   4. Disagree
   5. Strongly disagree
7. What did you find the most useful from participating in this session?
8. What would you change about the session going forward?
9. How do you plan to incorporate what you learned to your future clinical encounters?
